# *CACNA1D* overexpression and voltage-gated calcium channels in prostate cancer during androgen deprivation

**DOI:** 10.1038/s41598-023-28693-y

**Published:** 2023-03-22

**Authors:** Niamh McKerr, Adone Mohd-Sarip, Hannah Dorrian, Conor Breen, Jacqueline A. James, Stephen McQuaid, Ian G. Mills, Karen D. McCloskey

**Affiliations:** 1 Patrick G Johnston Centre for Cancer Research, School of Medicine, Dentistry and Biomedical Sciences, Queen’s University Belfast, 97 Lisburn Road, Belfast, Northern Ireland BT9 7AE UK; 2 Centre for Cancer Biomarkers (CCBIO), University of Bergen, Bergen, Norway; 3 Nuffield Department of Surgical Sciences, John Radcliffe Hospital, University of Oxford, Headley Way, OX3 9DU UK

**Keywords:** Cancer, Cell biology, Physiology

## Abstract

Prostate cancer is often treated by perturbing androgen receptor signalling. *CACNA1D*, encoding Ca_V_1.3 ion channels is upregulated in prostate cancer. Here we show how hormone therapy affects *CACNA1D* expression and Ca_V_1.3 function. Human prostate cells (LNCaP, VCaP, C4-2B, normal RWPE-1) and a tissue microarray were used. Cells were treated with anti-androgen drug, Enzalutamide (ENZ) or androgen-removal from media, mimicking androgen-deprivation therapy (ADT). Proliferation assays, qPCR, Western blot, immunofluorescence, Ca^2+^-imaging and patch-clamp electrophysiology were performed. Nifedipine, Bay K 8644 (Ca_V_1.3 inhibitor, activator), mibefradil, Ni^2+^ (Ca_V_3.2 inhibitors) and high K^+^ depolarising solution were employed. *CACNA1D* and Ca_V_1.3 protein are overexpressed in prostate tumours and *CACNA1D* was overexpressed in androgen-sensitive prostate cancer cells. In LNCaP, ADT or ENZ increased *CACNA1D* time-dependently whereas total protein showed little change. Untreated LNCaP were unresponsive to depolarising high K^+^/Bay K (to activate Ca_V_1.3); moreover, currents were rarely detected. ADT or ENZ-treated LNCaP exhibited nifedipine-sensitive Ca^2+^-transients; ADT-treated LNCaP exhibited mibefradil-sensitive or, occasionally, nifedipine-sensitive inward currents. *CACNA1D* knockdown reduced the subpopulation of treated-LNCaP with Ca_V_1.3 activity. VCaP displayed nifedipine-sensitive high K^+^/Bay K transients (responding subpopulation was increased by ENZ), and Ni^2+^-sensitive currents. Hormone therapy enables depolarization/Bay K-evoked Ca^2+^-transients and detection of Ca_V_1.3 and Ca_V_3.2 currents. Physiological and genomic *CACNA1D*/Ca_V_1.3 mechanisms are likely active during hormone therapy—their modulation may offer therapeutic advantage.

## Introduction

Prostate cancer (PCa) is the second leading cause of cancer death in UK males with around 11,900 deaths in 2018 (Cancer Research UK, https://www.cancerresearchuk.org/health-professional/cancer-statistics/statistics-by-cancer-type/prostate-cancer#heading-One,). PCa is a heterogeneous disease with inter- and intra-tumoral molecular complexity^1,2^ associated with genomic aetiology factors e.g. hereditary PCa and mutational status^3,4^ in addition to microenvironmental factors including inflammation and hypoxia^5,6^. Early localised PCa is commonly treated with surgery i.e. radical prostatectomy or radiotherapy with/without hormone therapy. Recurrent tumours are targeted with hormone therapy which includes androgen deprivation therapy (ADT, achieved through luteinising-hormone-releasing-hormone agonists and antagonists) or anti-androgen drugs (e.g. Enzalutamide, ENZ). Many PCa tumours eventually become resistant resulting in “castrate-resistant PCa” (or CRPC) which has poor survival outcomes.

Ca^2+^-signalling, normally understood in the context of cell physiology relating to muscle contraction, neurotransmission, hormone secretion and gene transcription, is known to be altered in cancer cell biology. Aberrant Ca^2+^-signalling in cancer cells has been linked to the cancer hallmarks of proliferation, invasion and migration^7,8^. Re-wiring of Ca^2+^-processes during malignant progression has been attributed to atypical expression and function of ion channels, pumps and transporters that may drive disease progression or treatment resistance^9,10^. These include voltage-gated Ca^2+^ channels (Ca_V_ family) which are Ca^2+^-permeant ion channels, activated in response to membrane depolarisation to facilitate Ca^2+^-influx.

Ca_V_ channels have three subfamilies: high voltage-activated, dihydropyridine-sensitive L-type (Ca_V_1.1–Ca_V_1.4); high voltage-activated dihydropyridine insensitive (Ca_V_2.1–Ca_V_2.3); and low-voltage activated T-type (Ca_V_3.1–Ca_V_3.3)^11,12^. Ca_V_ channels comprise pore-forming α1 subunits and a number of accessory/regulatory β and α2δ subunits. Ca_V_1.3, encoded by *CACNA1D*, is reportedly significantly upregulated in cancers compared with related normal tissue^13^. Laboratory studies demonstrate contribution of Ca_V_1.3 to breast, prostate and colorectal cancer through canonical (ion transport) and/or non-canonical (genomic) mechanisms^14–16^.

Several lines of evidence point to *CACNA1D*/Ca_V_1.3 expression in PCa as having biological significance. Gene transcript and protein are consistently reported as being overexpressed in PCa vs normal prostate epithelium in tumour samples and cell line models^17–22^. The question of whether Ca_V_1.3 upregulation in PCa is related to canonical ion channel function is interesting, not least as calcium channel blockers (CCB) are clinically used to treat hypertension and other conditions and could potentially be repurposed in cancer treatment^23^. Nevertheless, epidemiological evidence does not clearly support benefits of CCB in incidence or outcomes of PCa, and studies are conflicting. Some report increased PCa risk with CCB use^24,25^, others report no association, and potentially a protective effect upon PCa incidence^26–28^. An earlier study found no association between CCB use and PCa progression-free survival, overall survival or tumour aggressiveness^29^.

The molecular phenotype of PCa may provide insights into CCB use and disease risk. Geybels et al.^30^ concluded that the use of CCBs (ever vs. never) was not associated with overall PCa risk; however, CCB users who were European-American men had reduced risk of higher-grade PCa (Gleason scores ≥ 7). Furthermore, CCB use was associated with reduced risk of *TMPRSS2:ERG* positive (T2E^+^), which represents fusion of the androgen-regulated gene, *TMPRSS2* and the oncogene *ERG*, but CCB use was not associated with T2E^-^ PCa.

The present study established overexpression of Ca_V_1.3 in human PCa patient tissue and upregulation of *CACNA1D* in PCa cells, which was further enhanced in androgen-deprivation conditions or by ENZ. These conditions favoured switch to a neuroendocrine-like phenotype and detection of Ca_V_1.3-mediated Ca^2+^-transients in addition to recording of voltage-gated Ca^2+^-currents. The findings suggest that during PCa treatment, Ca_V_1.3 channels may contribute to Ca^2+^-physiology underpinning intracellular signalling.

## Materials and methods

### Cell culture

RWPE-1 (CRL-11609), LNCaP (CRL-1740), VCaP (CRL-2876), and PC3 (CRL-1435) were obtained from ATCC. C4-2, C4-2B and DU145 were a gift from the LaBonte Wilson group, Queen’s University Belfast. Cells were cultured in recommended growth medium supplemented with 10% FBS/1% penicillin–streptomycin and maintained at 37 °C/5% CO_2_. To mimic ADT, LNCaP were cultured in charcoal-stripped FBS-supplemented culture medium (CSFBS), or, treated with Enzalutamide (10 µM, ENZ, s1250, Selleck Chemicals) diluted in standard FBS-containing medium. Mycoplasma testing was performed bi-annually (MycoAlert, LT07-217, Lonza).

### Immunohistochemistry

Sections from a PCa tissue microarray (TMA) including normal and tumour tissues from 75 primary PCa patients were obtained from the Northern Ireland Biobank^31^ (Belfast, UK). In construction of the TMA^32^, three representative specimens for each subject were obtained from areas morphologically defined as “normal” and “tumour”, i.e., 6 specimens per subject; 450 cores (225 tumour, 225 normal). Tissue sections (4 µm) were processed for immunohistochemistry using an immunostainer (Leica Bond-Max, Milton Keynes, UK). Protocols were optimised for each antibody with the following final dilutions: anti-Ca_V_1.3 (1:75, rabbit polyclonal, HPA020215, Sigma-Aldrich, UK) preceded by antigen retrieval solution 2 (10 min), and anti-CK5/6 (1:50, mouse monoclonal, clone no. D5/16 B4, Dako, Cambridgeshire, UK) preceded by antigen retrieval solution 1 (30 min). Positive CK5/6 staining is typically found on benign glands, enabling verification of tumour areas^33^. Labelled sections were visualised with diaminobenzidine (DAB) and haematoxylin.

TMA slides were independently scored using a Q-score system by NMcK and KMcC and verified by pathologists (JJ and SMcQ) through staining intensity (0, 1, 2, 3) times the percentage of epithelial tissue comprising that intensity. Assessors were blinded to clinical features and outcome. After quality control criteria were applied to the stained cores, 67 cases represented by approximately 402 cores (201 each tumour and normal) were feasible for Ca_V_1.3 evaluation and were analysed. Maximum intensity Q score across each set of triplicates were evaluated.

### qPCR

Total RNA was extracted from cells using Tripure isolation reagent as per the Manufacturer’s protocol (Roche, Cat No. 11667165001) and quantified using a NanoDrop spectrophotometer (ND-1000, Thermofisher Scientific). RNA purity 260/280 nm and 260/230 nm absorbance ratios were confirmed to be within acceptable limits (1.8–2.0 and 2.0–2.2 respectively). Reverse transcription was performed on RNA (1 µg) using the Roche Transcriptor High Fidelity cDNA Synthesis Kit (Sigma-Aldrich-Aldrich, 5081963001). Primers were designed using The Universal ProbeLibrary from Roche (Roche Life Science, UK), sequences are presented in Table 1.

**Table 1:** qPCR primers and sequences.

Primer	Sequence
*CACNA1D* forward	5′-AAGGACCAACTTCTCAGCCG-3′
*CACNA1D* reverse	5′-TGCTCTTGGCGTATTGCTGA-3′
*RPLPO* forward	5′-AATCTCCAGGGGCACCATT-3′
*RPLPO* reverse	5′-CGCTGGCTCCCATTTGT-3

The qRT-PCR reaction was performed using a LightCycler 480 PCR system (Roche). Each assay was performed using triplicate measurements where relative transcript levels were normalised against reference gene, *RPLPO* (Table 1) using the 2−ΔΔCT relative quantification method.

### Western blotting

Cells were lysed in RIPA buffer for 30 min on ice and centrifuged at 13,800*g* for 15 min, 4 °C. Lysates were quantified using protein quantification reagent (500–0006, Bio-Rad), prepared using Laemmli 2 × sample buffer (Sigma-Aldrich), for 5 min, 95 °C. Whole cell lysates (30 μg) were resolved on polyacrylamide gels then transferred to nitrocellulose membranes. Membranes were blocked with Tris-buffered saline containing 5% milk, 22 °C, 1 h and incubated with primary antibodies overnight at 4 °C (Table 2). Pre-absorption of anti-Ca_V_1.3 with the control peptide was carried out to test specificity and molecular weight validation, (Alomone protocol). Membranes were incubated with HRP-linked secondary antibodies, 22 °C for 1 h. Specific proteins were visualised using Immobilin Crescendo HRP Substrate (WBLUR0500, Merck). To maximise protein investigation, blots were sometimes cut to enable hybridisation with a number of primary and secondary antibodies. The supplementary figures present the entire experiment i.e. all cut blots that comprised a full blot so that membrane edges and the size of protein bands are visible. Proteins that were investigated but were not relevant to the present paper are indicated as ‘undisclosed’. Imaging was performed on a Syngene G:Box Chemi XX6 system and GeneSys imaging software (v1.4.6.0). Merged images presented in Supplementary figures show the membrane boundary. These are an overlay of the chemiluminescent image on the colorimetric molecular weight ladder image; this merging of the two images occurs automatically on the GeneSys imaging software.

**Table 2:** Western blotting and IF antibodies.

Antibody	Dilution/species	Supplier (product no.)
Ca_V_1.3 (extracellular)	1:300 (WB), 1:200 (IF), Rabbit	Alomone (ACC-311)
Neuron specific enolase (NSE)	1:1000 Mouse (WB)	Abcam (85F11)
Androgen receptor (AR)	1:1000 Mouse (WB)	Abcam (ab9474)
GAPDH	1:20,000, Mouse (WB)	Bio-rad (MCA4740)
Anti-rabbit IgG, HRP-linked	1:6000 (WB)	Cell signalling (7074S)
Anti-mouse IgG, HRP-linked	1:6000 (WB)	Cell signalling (7076S)
Anti-rabbit IgG (H + L), donkey Alexa Fluor 488	1:400 (IF)	Thermofisher scientific (A-21206)

### Immunofluorescence

Cells were seeded overnight on PDL-coated coverslips. Cells were fixed (4% PFA), permeabilised (0.5% saponin) and blocked (0.3 M Glycine/1% BSA) then incubated in anti-Ca_V_1.3 (Table 2) overnight at 4 °C. After washing in PBS, cells were incubated with secondary antibody and DAPI (200 ng/ml, D9542, Sigma-Aldrich). Secondary antibody controls were conducted by omission of primary antibody. Image acquisition was performed using a Leica TCS SP8 confocal scanning microscope (Leica Microsystems (UK) Ltd, Milton Keynes, UK) on an inverted DMi8 microscope using an oil objective (HC PL APO CS2, 63x, 1.40 NA) and Leica Application Suite X software (3.5.7.23225). Images were collected as z-series and the micrographs presented here are single optical sections, approximately mid-way through the cell depth.

### Brightfield microscopy

Images were obtained using an EVOS XL Core Imaging System (ThermoFisher Scientific). Five images were captured for each condition at specified time-points. Image J software^34^ was used to measure neurite length, defined as extending neurites between the cell body of neighbouring cells (measured in 2 cell pairs per image, 10 measurements per biological replicate).

### Ca^2+^ imaging

Cells were seeded in PDL-coated culture dishes (ThermoFisher Scientific, Nunc) and loaded with Cal 520-AM (10 µM, 21130, AAT-Bioquest) for 90 min or with Fluo-4 AM (2 µM F14201, ThermoFisher Scientific, and probenecid, 2 mM, Sigma-Aldrich) for 1 h at 37 °C, then perfused with Hanks’ Physiological Saline Solution for 20 min by a gravity-perfusion system (1 ml min^−1^). Drugs were added via the perfusion.

Solutions used comprised (mM): (1) Hanks’ Physiological Saline Solution (PSS): 125 NaCl, 5.4 KCl, 10 glucose, 2.9 sucrose, 4.2 NaHCO_3_, 0.4 KH_2_PO_4_, 0.3 NaH_2_PO_4_, 0.5 MgCl_2_·6H_2_O, 1.8 CaCl_2_·2H_2_O, 0.4 MgSO_4_, 10 HEPES (free acid), pH 7.4, osmolarity calculated as 301.2 mOsm/L. (2) High K^+^ PSS (for depolarization) 70.8 NaCl, 59.65 KCl, 10 glucose, 2.9 sucrose, 4.2 NaHCO_3_, 0.4 KH_2_PO_4_, 0.3 NaH_2_PO_4_, 0.5 MgCl_2_·6H_2_O, 1.8 CaCl_2_·2H_2_O, 0.4 MgSO_4_, 10 HEPES, pH 7.4, 301.3 mOsm/L.

Cells were imaged with a Nikon Eclipse 80i upright epifluorescent microscope (Nikon, Surrey, UK) equipped with an Andor Zyla 4.2 sCMOS camera (Oxford Instruments, UK) using a 10x water-dipping objective lens (CFI Plan Fluor 10x/0.30 W) and WinFluor software (v.4.0.3, Strathclyde University). Recordings were made for 400 s, frame-rate 5 frames per second utilising a 2 × 2 binning function in WinFluor. The Ca^2+^-ionophore, Ionomycin (1 µM, Abcam) was added as a positive control at the end of each recording.

Regions of interest (ROI) were placed on all “responsive” (see below) cells in a field of view (FOV) within each recording (up to 750 cells per FOV). An additional ROI was placed in a cell-free area for subtraction of background fluorescence. The background-corrected fluorescence (F) at any given time was normalised to baseline (F_0_), where F_0_ was the mean fluorescence in each ROI, during 20 frames at the beginning of the recording where no activity was present. Recordings are presented as intensity time-plots where intensity was calculated as ΔF/F_0_ = (F − F_0_)/F_0._ A “responsive” cell met the following criteria: (i) fluorescence exceeded a threshold ΔF/F_0_ ≥ 0.2; (ii) the Ca^2+^-transient occurred within 30–100 s post-drug perfusion; and (iii) had a distinct rise in fluorescence, unlikely to be complicated with any background oscillations. Fluorescence intensity measurements were made in WinFluor and analysed in Microsoft Excel and Prism software (v5.03 or v9.5.1, Graphpad). Measurements of amplitude from all ROIs in a recording were averaged to produce an N = 1.

### Proliferation assay

Cells were seeded overnight in PDL-coated plates and left to adhere. Nifedipine (0–100 µM) treatments were performed in triplicate over 72 h. PrestoBlue cell viability reagent was added and incubated at 37 °C. A BioTek Synergy 4 plate reader was used to determine the fluorescence intensity of each well at 560 nm and 590 nm. Readings were analysed using Microsoft Excel where the mean was calculated from triplicate readings and compared against respective controls. Statistical analysis details are indicated in the relevant figure.

### Patch clamp electrophysiology

Cells were patch-clamped in whole-cell mode using Cs^+^-filled pipettes as previously described^35^. The following ion channel modulators were used: Tetraethylammonium chloride (TEA, T2265, Sigma-Aldrich) to block dominant BK channels, Nifedipine (N7634, Sigma-Aldrich) to inhibit Ca_V_1.3 channels, Bay K 8644 (1544, Tocris) to activate Ca_V_1.3 channels and Mibefradil (2198, Tocris) or NiCl_2_ (Ni^2+^) to block Ca_V_3.2 channels. Nifedipine and Bay K 8644 were prepared as stocks in DMSO and diluted so that the final vehicle concentration was < 0.1%. Cs^+^-pipette solution comprised (mM): 110 CsCl, 20 TEA-Cl; 5 MgCl_2_; 1 EGTA; 0.1 Na_2_GTP; 2.5 Na_2_phosphocreatine; 4 Mg-ATP; 5 HEPES, 296.8 mOsm/L, pH 7.2. TEA-Ba^2+^ PSS comprised (mM): 115 NaCl, 10 TEA-Cl, 1 MgCl_2,_ 5.5 Glucose, 10 BaCl_2_, 10 HEPES, 298.5 mOsm/L, pH 7.4. Divalent-free PSS comprised (mM): 129.8 NaCl, 5.4 KCl, 10 glucose, 2.9 sucrose, 4.2 NaHCO_3_, 0.4 KH_2_PO_4_, 0.3 NaH_2_PO_4_, 5 EGTA, 10 HEPES, osmolarity 308.1 mOsm/L, pH 7.4.

### siRNA *CACNA1D* knockdown

After 11 days CSFBS culturing, reverse transfection was performed with Lipofectamine RNAiMax (Invitrogen, Thermofisher) according to the Manufacturer’s instructions. Cells were transfected with 50 nM scrambled control (5′-UAAUGUAUUGGAACGCAUA-3′, Eurofins) or siRNA targeting *CACNA1D* (L-006124-00-005, ON-TARGETplus SMARTpool, Dharmacon) in OptiMEM reduced serum medium (Gibco, Thermofisher). At 18–24 h, dishes were replenished with fresh culture medium. Cells were harvested or used for Ca^2+^-imaging 72 h post-transfection (14 day time-point).

### Statistical analysis

**Figure 1:**
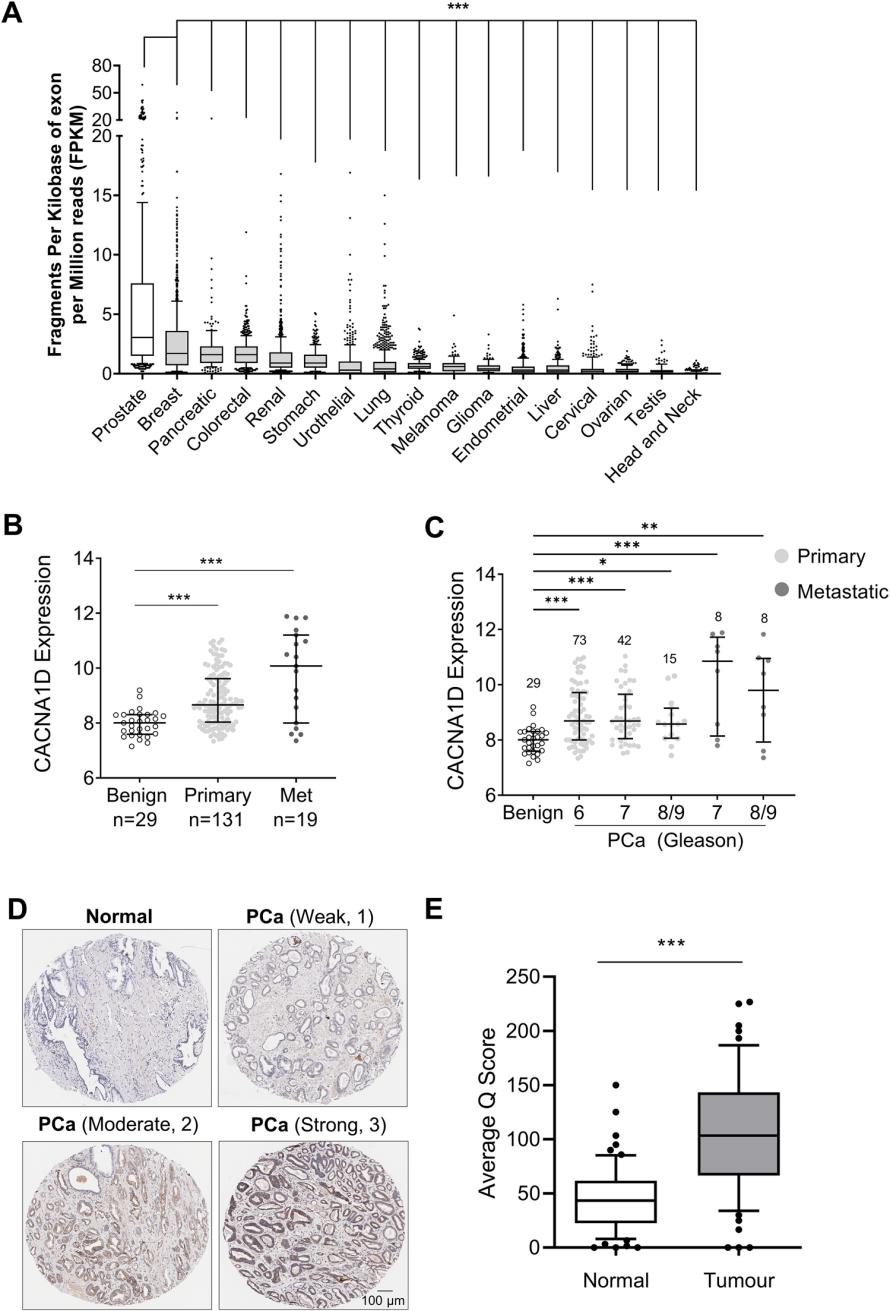
Profiling *CACNA1D* and Ca_V_1.3 expression in clinical PCa datasets and tissue microarrays. (**A**) Human *CACNA1D* transcript expression was determined by RNA-seq analysis from 7932 TCGA samples from 17 different cancer tissues. Data was obtained from The Human Protein Atlas (www.proteinatlas.org/) ENSG00000157388-CACNA1D/pathology (v21.1.proteinatlas.org), is presented as box-and-whisker plots (median, 10–90 percentile) and was analysed with Kruskal–Wallis, Dunn’s multiple comparison test p < 0.001 (***). (**B**) Scatter plot showing *CACNA1D* expression in primary (n = 131) PCa and metastatic PCa (n = 19) compared to adjacent benign prostate tissue (n = 29). Data was derived from GEO dataset, GSE21032 (GPL 5188) conducted by Memorial Sloan-Kettering Cancer Centre (MSKCC). Data is expressed as median and inter-quartile range and was analysed using Kruskal–Wallis, Dunn’s multiple comparison test, p < 0.001 (***). **C**. *CACNA1D* expression was compared by Gleason grade (6, 7 and 8–9) between primary and metastatic PCa samples. Data was derived from GEO dataset, GSE21032 (GPL 5188, MSKCC) and analysed using Kruskal–Wallis, Dunn’s multiple comparison test. Data is expressed as median and inter-quartile range where significance is indicated as p < 0.05 (*), p < 0.01 (**) and p < 0.001 (***). D. **E**xamples of normal/benign and prostate tumour tissue cores from an in house tumour PCa microarray, stained with anti-Ca_V_1.3 (HPA020215, Sigma Aldrich). Tissues were assessed by a Q scoring system. Staining intensity was scored as weak (1), moderate (2) and strong (3) and was multiplied by the percentage of tissue encompassed by the designated staining. (**E**) Q scores (median, 10–90 percentile) showing significant Ca_V_1.3 overexpression in tumour vs normal/benign tissues (N = 67, Mann–Whitney, unpaired, p < 0.001 (***)).

**Figure 2:**
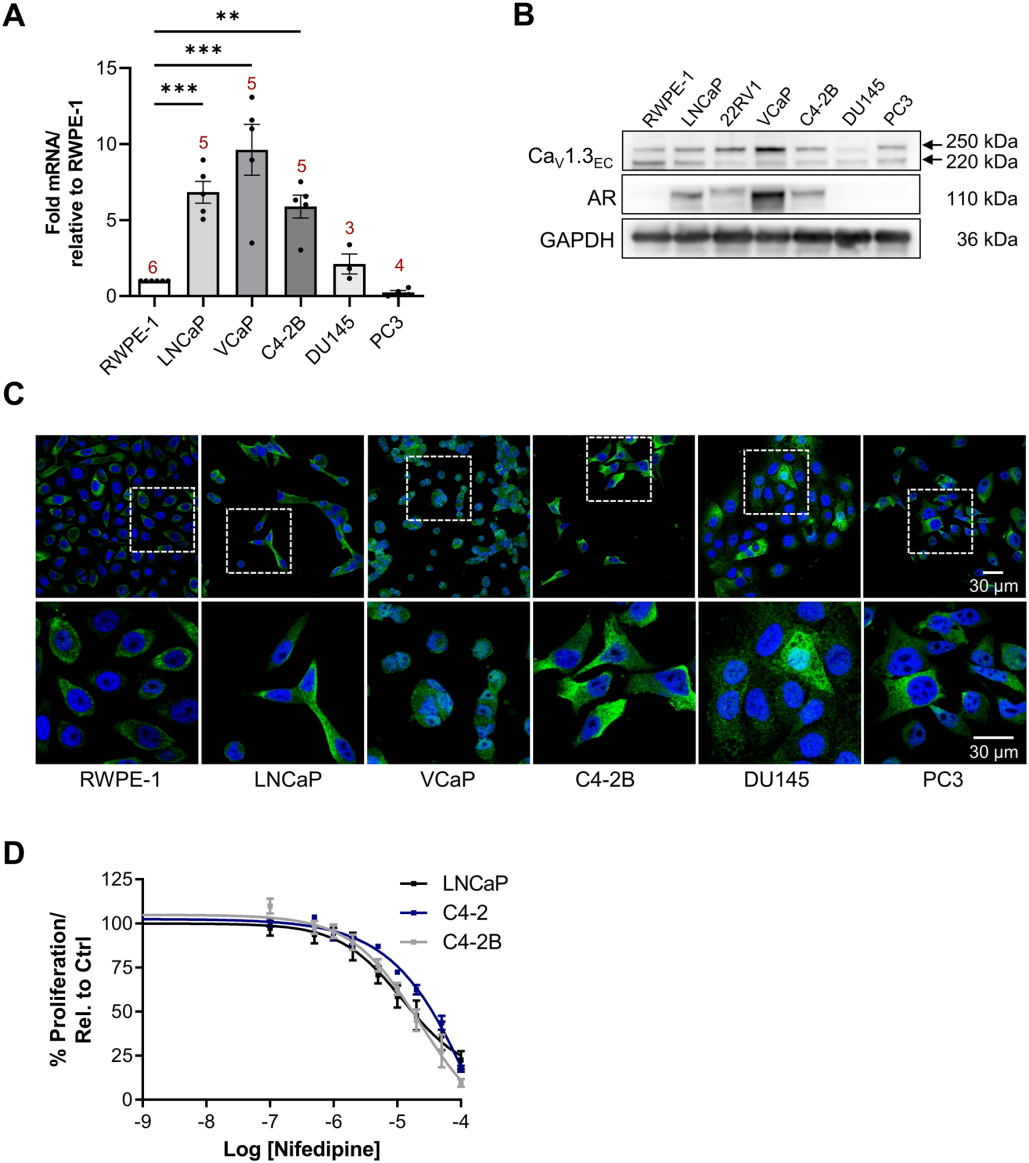
Ca_V_1.3 expression profiling using in vitro PCa cell lines. (**A**) qPCR was performed to assess CACNA1D transcript expression in normal and PCa cell lines. RPLPO was used as an endogenous reference control. Fold changes were determined by normalisation to RPLPO and RWPE-1 transcript levels. Data was analysed using 1-way ANOVA (Dunnett’s) where p < 0.01 (**), p < 0.001 (***) comparing PCa cells to RWPE-1 (N indicated above each bar). (**B**) Protein expression of Ca_V_1.3 and androgen receptor, AR was analysed in the panel of cell lines in Western blots with GAPDH as a loading control (N = 3). Uncropped original blots are displayed in Supplementary Fig. S4. (**C**) Immunofluorescence micrographs of Ca_V_1.3 (green) and nuclear staining by DAPI (blue) for each cell line (N = 3). White boxes in the top row of micrographs are shown at higher magnification in the bottom row. (**D**) Summary data (mean, SEM) of proliferation across LNCaP, C4-2 and C4-2B treated with nifedipine (0–100 µM) for 72 h (N = 3) showing concentration-dependent decrease in proliferation.

**Figure 3:**
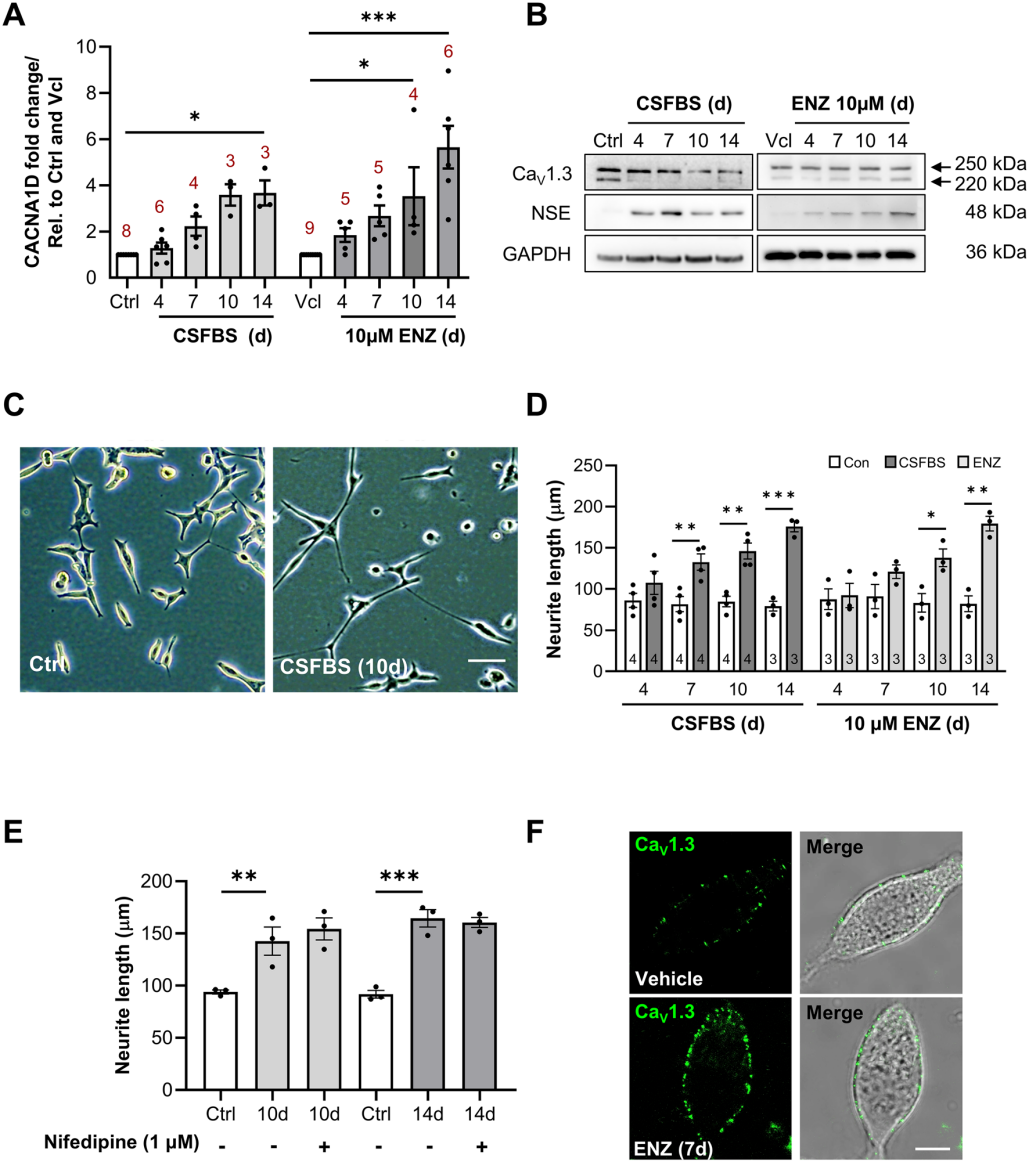
Changes in *CACNA1D* and Ca_V_1.3 expression during recapitulation of hormone therapy. (**A**) *CACNA1D* transcript expression in CSFBS-cultured and ENZ-treated LNCaP. Transcription expression was normalised to control/vehicle *CACNA1D* expression and *RPLPO* housekeeping gene (N indicated above the bars, ‘d’ denotes days). Data was analysed with Kruskal–Wallis test (Dunn’s multiple comparison), p < 0.05 (*) or p < 0.001 (***). (**B**) Western blotting micrographs showing Ca_V_1.3 and NSE expression during CSFBS-culturing and ENZ treatment (4–14 days, N = 3). GAPDH was used as a loading control. Uncropped original blots are displayed in Supplementary Figs. S5 and S6. (**C**) Representative light micrographs showing neurite-like extensions in CSFBS-cultured LNCaP compared to untreated controls at 10 days. Scale bar (white line) = 100 µm. (**D**) Average neurite length (µm) in time controls and CSFBS-cultured LNCaP, and during ENZ were compared. Data was analysed using unpaired t-tests with Welch’s correction where p < 0.05 (*), p < 0.01 (**), p < 0.001 (***) (N indicated within bars). (**E**) Nifedipine had no effect on neurite length (µm) in 10 day and 14 day CSFBS-cultured LNCaP. Data was analysed using 1-way ANOVA (Šídák's multiple.comparison test) where p < 0.01 (**) and p < 0.001 (***) (N = 3). (**F**) Ca_V_1.3 membrane surface expression was assessed in live-labelled, non-permeabilised cells imaged with confocal microscopy. Visible Ca_V_1.3 membrane foci were observed on the plasma membrane of both vehicle and ENZ-treated (7 days) LNCaP cells (N = 3). Scale bar (white line) = 10 μm.

**Figure 4:**
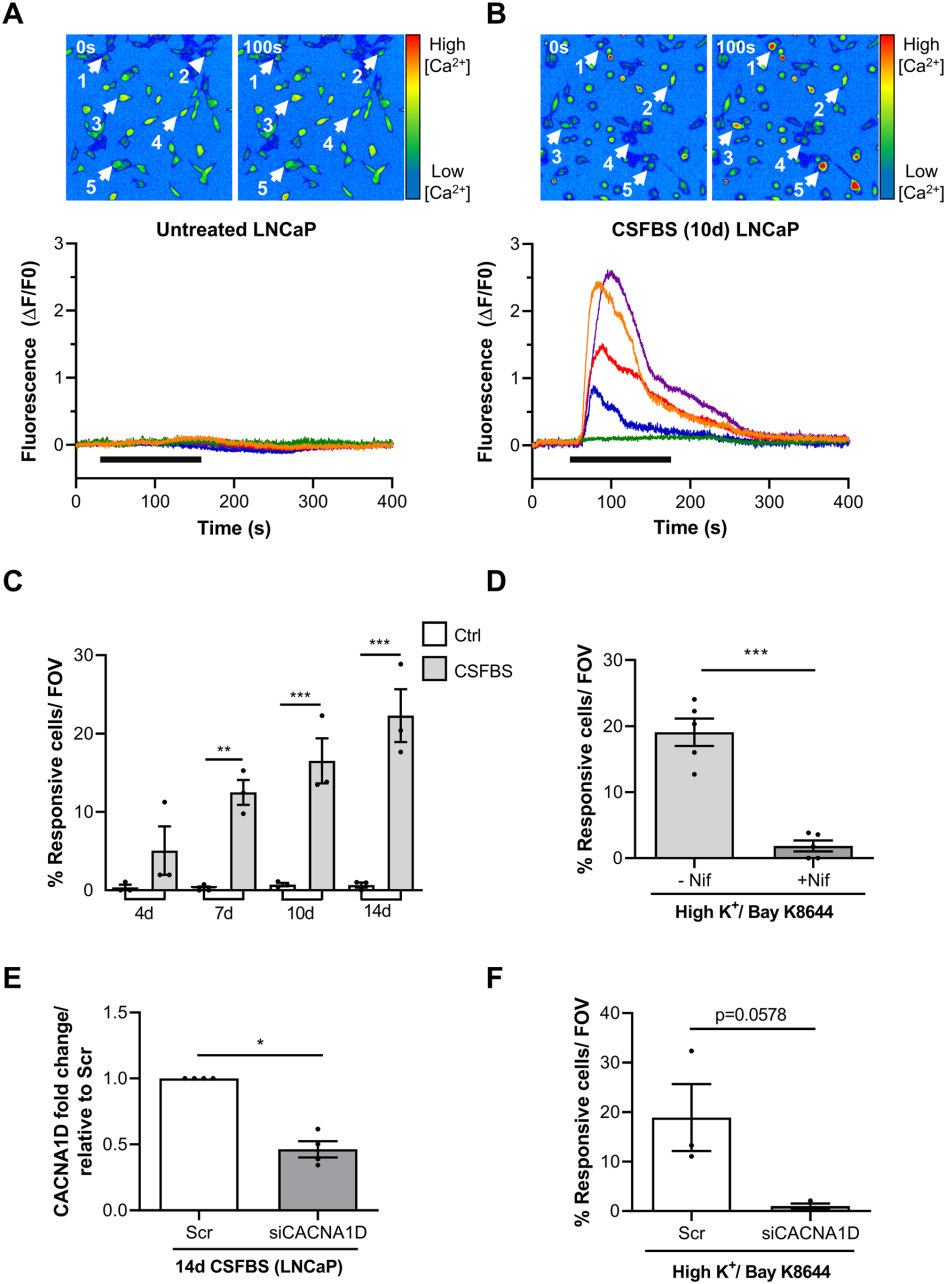
Ca_V_1.3-mediated Ca^2+^ signalling emerges in LNCaP during CSFBS culturing. (**A**) Pseudocolour micrographs of Cal-520 AM-loaded LNCaP (cultured in media containing normal FBS) during live-cell recordings. Pseudocolour scale indicating increased fluorescence intensity from blue–green–yellow–red, indicating increased intracellular [Ca^2+^]. Fluorescence micrographs in normal Hanks' (0 s) and during perfusion with high K^+^ Hanks containing Bay K 8644 (10 µM, denoted by solid horizontal bar) (100 s) are shown. Five cells (1–5, white arrows) were selected and ΔF/F0 measured then plotted on a fluorescence intensity-time graph (coloured lines, lower panel) (N = 5 recordings). (**B**) Pseudocolour micrographs showing CSFBS-cultured LNCaP (10 days) pre- (0 s) and during exposure (100 s) to high K^+^/Bay K 8644. Fluorescence intensity of 5 selected cells demonstrating Ca^2+^-transients in 4 of the 5 cells (lower panel) (N = 3 recordings). (**C**) Mean percentage of responding cells per recording is shown at several time points during CSFBS along with matched time controls. Data analysed with 1-way ANOVA with Šídák's multiple comparison test, p < 0.05 (*), p < 0.01 (**) and p < 0.001 (***) (N = 3). (**D**) Nifedipine (1 µM) significantly reduced the percentage of high K^+^/Bay K 8644-responding cells after 10 day CSFBS. Data was analysed with unpaired t-test where p < 0.001 (***) (N = 5 recordings). (**E**) Significant knockdown of CACNA1D by siRNA transfection was achieved in CSBFS-treated LNCaP (14 days, Mann–Whitney, N = 4, p < 0.05 (*). (**F**) The percentage of high K^+^/Bay K8644-responsive LNCaP in siRNA-LNCaP was markedly reduced compared with scrambled controls, (p = 0.0578, unpaired t-test, N = 3).

**Figure 5:**
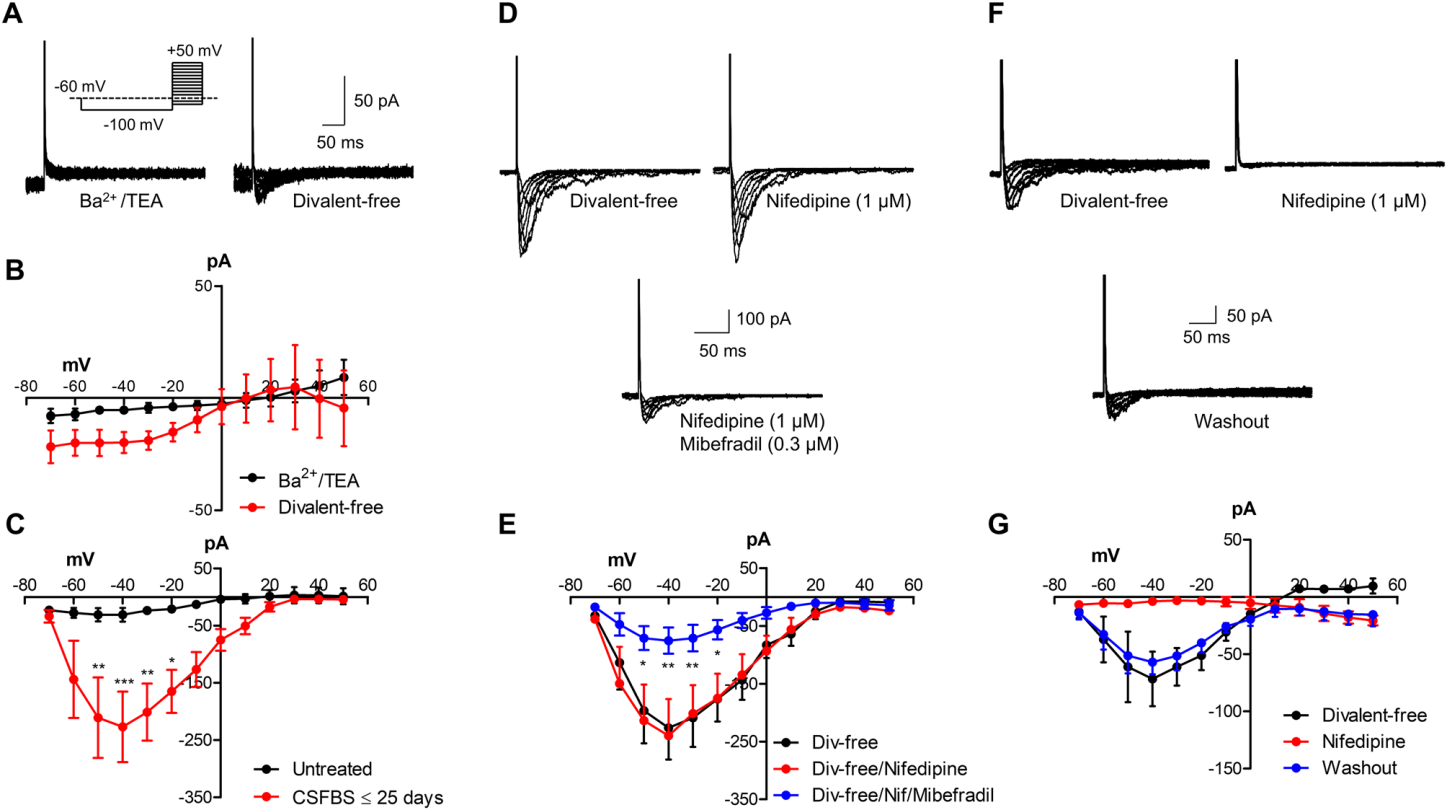
Voltage-gated inward currents in LNCaP emerge after CSFBS culturing. (**A**) Representative recordings from untreated LNCaP in Ba^2+^/TEA Hanks, and detection of inward currents in divalent-free solution. (**B**) Summary current–voltage plot of mean current from 6 cells (N = 2 cultures) in BaLNCaP exhibited high K/TEA and divalent free conditions. (**C**) Summary current–voltage plot of divalent-free currents in untreated LNCaP (n = 8, N = 2 cultures, overlapping with n = 6 data from panel **B**) and CSFBS-LNCaP up to 25 days treatment (n = 11, N = 4). Two-way ANOVA, Bonferroni post-hoc tests showed significant increase in mean current at − 50, − 40, − 30 and − 20 mV (*p < 0.05, **p < 0.01; ***p < 0.001). (**D**) Representative currents in divalent-free from an LNCaP cultured in CSFBS or 25 days in the presence of nifedipine and upon further addition of mibefradil. (**E**) Summary current–voltage plot for the effect of nifedipine (1 μM) alone and combined with (0.3 μM mibefradil) in CSFBS treated cells (n = 6; N = 4) where mibefradil significantly reduced current amplitude. (**F**) In CSFBS treated cells (14 days), nifedipine-sensitive currents were less frequently encountered and the effect of nifedipine was largely reversible. (**G**) Summary current–voltage plot for 3 cells from one culture that exhibited nifedipine-sensitive inward currents.

**Figure 6:**
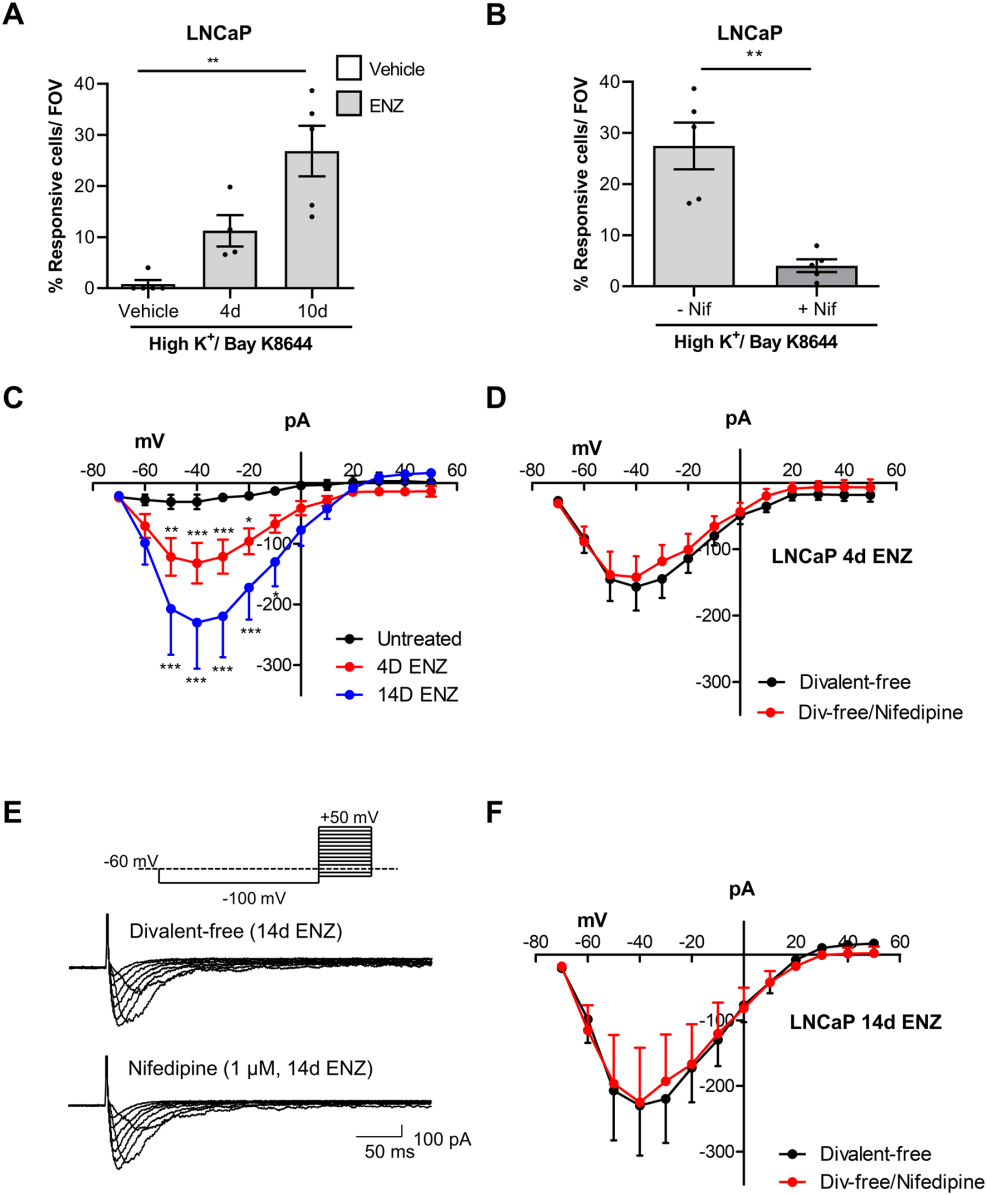
Ca_V_-mediated Ca^2+^ signalling in LNCaP after Enzalutamide treatment. (**A**) LNCaP exhibited high K^+^/Bay K8644-Ca^2+^ -transients after ENZ for 4 days or 10 days; Kruskal–Wallis with Dunn’s multiple comparison test, p < 0.01 (**) (N = 3). (**B**) The percentage of cells, treated with ENZ for 10 days, responding to the stimulus was significantly reduced in the presence of nifedipine (1 µM, unpaired t-test, p < 0.01 (**) (N = 3). Note, data for 10 day ENZ in the absence of nifedipine is replotted from panel (**A**). (**C**) Summary current–voltage plot of divalent-free currents in untreated, ENZ-LNCaP 4 days and 14 days treatment (n = 8, N = 2 cultures; n = 11, N = 4 cultures; n = 5, N = 2 cultures respectively). Two-way ANOVA, Bonferroni post-hoc tests showed significant increase in mean current between untreated vs 4 days ENZ, or untreated vs 14 days ENZ (*p < 0.05, **p < 0.01; ***p < 0.001). (**D**) Summary current–voltage plot for ENZ-LNCaP (4 days treatment) (n = 9 cells, N = 4 cultures) currents recorded in divalent-free before and after nifedipine (1 μM). (**E**) Representative recordings from ENZ-LNCap (14 days) in divalent-free and in nifedipine. (**F**) Summary current–voltage graph showing currents in absence and presence of nifedipine, ENZ-LNCaP after 14 days treatment (n = 5 cells, N = 2 cultures).

**Figure 7:**
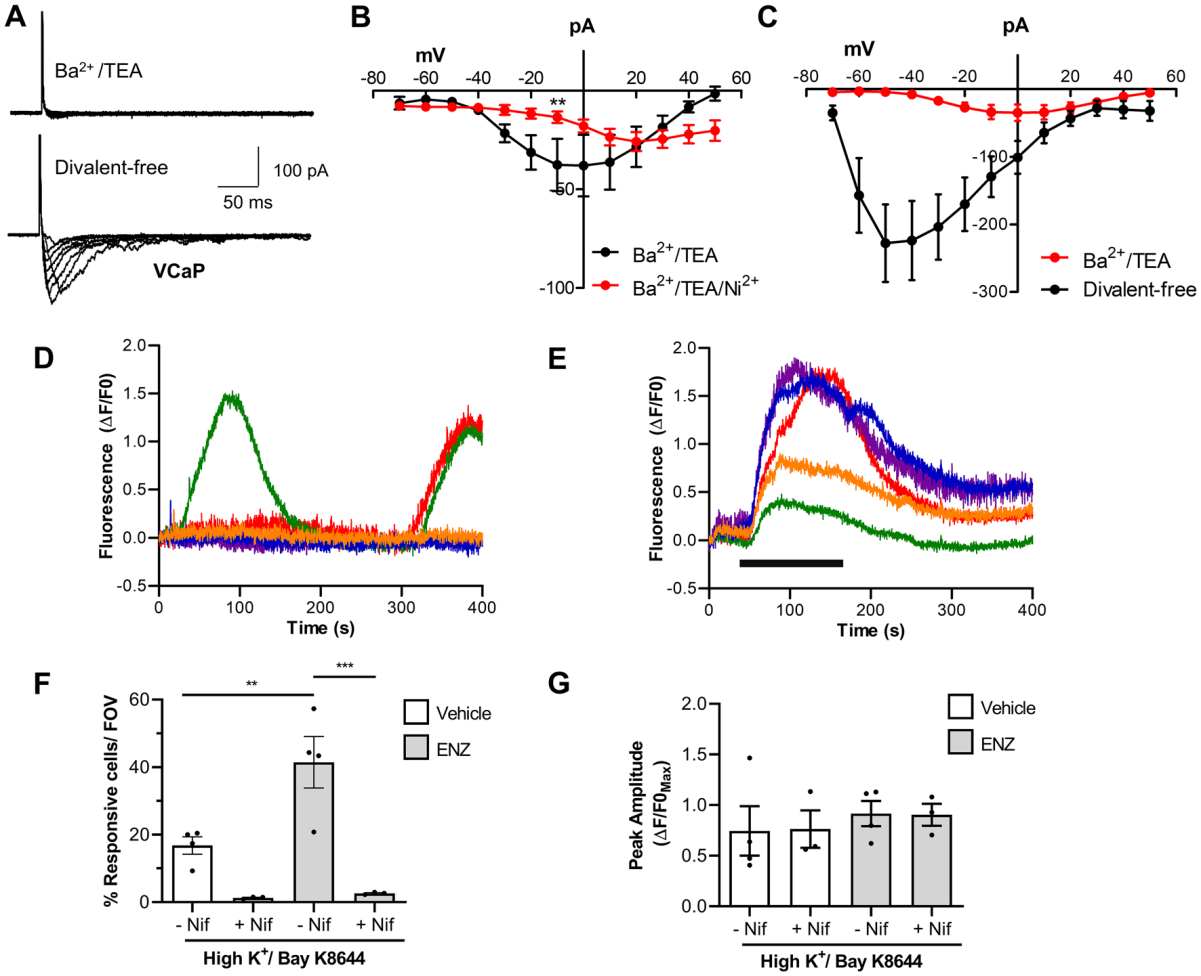
Ca_V_-mediated Ca^2+^ signalling in T2E^+^ VCaP. (**A**) Representative currents from VCaP in Ba^2+^ /TEA Hanks and in divalent-free solution. (**B**) A portion of the inward current was Ni^2+^-sensitive; the residual current–voltage plot was right-shifted by 20 mV (n = 7 cells, N = 5 cultures). (**C**) Summary current–voltage plot for VCaP currents (n = 9 cells, N = 7 cultures) recorded in Ba^2+/^TEA Hanks and in divalent-free solution. (**D**) Representative recordings from 5 VCaP perfused with Hanks. Oscillating cells (green and red) were typical of 2.9 ± 1.2% (mean ± SEM) VCaP within each field of view (N = 5 recordings). (**E**) VCaP were challenged with high K^+^/Bay K 8644 (10 µM) as indicated with a horizontal bar, and Ca^2+^ transients could frequently be recorded. Five ROI are plotted on a fluorescence-time graph (typical of N = 3 recordings). (**F**) ENZ significantly increased the percentage of high K^+^/Bay K 8644-responsive VCaP per recording compared with vehicle controls (1-way ANOVA with Šídák's multiple comparison test, p < 0.01 (**) or p < 0.001 (***), N = 4). (**G**) The average amplitude (ΔF/F0) of high K^+^/Bay K 8644-induced Ca^2+^events were similar in the absence and presence of ENZ or nifedipine.

Experimental data was analysed in Prism (v5.03 or v8.0, GraphPad) using appropriate parametric and non-parametric tests. In Fig. 1, data is expressed as mean (10–90 percentile) or median (inter-quartile range) where significance is p < 0.05. In Figs. 2, 3, 4, 5, 6 and 7, data is expressed as mean ± SEM where significance is p < 0.05. Statistical significance is expressed as p < 0.05 (*), p < 0.01 (**) and p < 0.001 (***). Statistical tests were selected after testing for normality; tests used and significance details are outlined in the figure legends.

## Results

RNA-sequencing data from The Cancer Genome Atlas (TCGA) was obtained from http://www.proteinatlas.org, a publicly available resource, facilitating large-scale transcriptomic and protein analyses from cells/clinical tissues. The *CACNA1D* RNA dataset www.proteinatlas.org/ENSG00000157388-CACNA1D/pathology (v21.1.proteinatlas.org) included 7932 TCGA samples from 17 cancer types^36,37^. *CACNA1D* expression was calculated using FPKM (Fragments Per Kilobase of exon per Million reads) where the detection threshold was pre-set to 1 FPKM. Box and whisker plots (median, 10th, 90th percentiles) demonstrated *CACNA1D* transcript abundance in each cancer type, arranged in descending mean FPKM order (Fig. 1A). Greatest mean *CACNA1D* expression occurred in PCa (FPKM:5.93) followed by breast (FPKM: 2.58), pancreatic (FPKM: 1.96) and colorectal (FPKM:1.75) cancers in agreement with previous studies^13,15,16^.

Publicly available data from the Memorial Sloan Kettering Cancer Centre (MSKCC) on Gene Expression Omnibus (GEO) was used to assess *CACNA1D* expression in PCa^38^ (Accession GSE21032, Platform GPL5188). Significant *CACNA1D* overexpression was found in primary and metastatic PCa tumours, compared to adjacent benign prostate tissue (Fig. 1B). The same data was subdivided by Gleason grade. Samples not containing information about Gleason Grade (had been denoted N/A) or had insufficient numbers for comparison (e.g. Gleason grade 6 in metastatic category) were excluded from the analysis. Significant *CACNA1D* overexpression was detected across all Gleason grades in primary and metastatic PCa (Fig. 1C). Ca_V_1.3 protein expression was examined in an in house PCa TMA (N = 75 subjects)^32^ CK5/6 staining in sequential sections enabled visualisation of benign vs tumour tissue (Supplementary Fig. S1). Ca_V_1.3-positive scoring was performed by 2 independent, blinded assessors and was independently validated by a pathologist. Staining intensity was graded as 0 (no staining), 1 (weak), 2 (moderate) or 3 (strong) (Fig. 1D). Ca_V_1.3 overexpression via Q-scoring was detected in PCa tissue compared to normal regions (N = 67; p < 0.001, Fig. 1E).

*CACNA1D* and Ca_V_1.3 expression was investigated in cell lines representing molecular phenotypes including; androgen sensitivity (LNCaP, 22RV1, VCaP (AR amplification), C4-2B), castrate-resistance (C4-2B, DU145, PC3); T2E^+^ gene fusion (VCaP) and metastatic phenotype (C4-2B, DU145 and PC3) and an immortalised epithelial prostate cell line, RWPE-1. *CACNA1D* overexpression was detected in AR-positive LNCaP (6.8 ± 0.7 fold, N = 5, p < 0.001), VCaP (9.6 ± 1.7 fold, N = 5, p < 0.001) and C4-2B (5.9 ± 0.8 fold, N = 5, p < 0.01 vs RWPE (N = 6, Fig. 2A). *CACNA1D* was notably lower in AR-negative, metastatic PCa DU145 (2.1 ± 0.7 fold, N = 3, p > 0.05) and PC3 (0.2 ± 0.1 fold, N = 4, p > 0.05) than RWPE-1 (N = 6).

Ca_V_1.3 was detected in all cell lines (Fig. 2B) with the expected 250 kDa band detected, in addition to a 220 kDa band. AR expression was confirmed in LNCaP, VCaP, 22RV1 and C4-2B; moreover, Ca_V_1.3 had highest expression in the AR-overexpressing and T2E^+^ VCaP (N = 3). Immunofluorescence showed cytoplasmic Ca_V_1.3 localisation across all cell lines (Fig. 2C). VCaP also had nuclear Ca_V_1.3 immunofluorescence. Antibody validation and relevant controls with pre-absorption of the primary antibody with its peptide control antigen and secondary-only controls were carried out in immunofluorescence experiments (Supplementary Fig. S2A). Western blots demonstrated specificity of the primary antibody at the expected 250 kDa band. Additional specific bands at approximately 220 and 75 kDa bands were not detected when the primary antibody was pre-incubated with control antigen (Supplementary Fig. S2B, N = 3). The identity of these bands have not been validated; however, they may account for splice variants or other post-translational modifications.

The Ca_V_1.3 channel blocker Nifedipine, was tested on proliferation of androgen-sensitive LNCaP, C4-2 and C4-2B (0–100 µM, 72 h, N = 3). Increasing concentrations of nifedipine were correlated with decreased proliferation (Fig. 2D). The GI_50_, (concentration resulting in 50% inhibition of proliferation), was 12.2 µM in LNCaP, 25.8 µM in C4-2 and 22.8 µM in C4-2B.

Given the Nifedipine-induced cytostatic effects on LNCaP and derivative cell lines, gene and protein expression was investigated in LNCaP undergoing recapitulation of hormone therapy (CSFBS or ENZ in FBS-containing media). CSFBS or ENZ increased *CACNA1D* mRNA expression over 14 days. *CACNA1D* was overexpressed in LNCaP during CSFBS (1.3 ± 0.2 fold [4d, N = 6], 2.2 ± 0.4 fold [7d, N = 4], 3.6 ± 0.5 fold [10d, N = 3] and 3.7 ± 0.5 fold [14d, p < 0.05, N = 3] compared with untreated cells (N = 8) (Fig. 3A). ENZ also caused overexpression (1.8 ± 0.3 fold [4d, N = 5], 2.7 ± 0.5 fold [7d, N = 5], 3.5 ± 1.7 fold [10d, p < 0.05, N = 4] and 5.6 ± 0.9 fold [14d, p < 0.001, N = 6]) compared to vehicle controls. Total Ca_V_1.3 protein (250 kDa) expression did not mirror the increase in *CACNA1D* observed over time and either showed little change during ENZ, or in CSFBS apparently decreased at 14 days. (Fig. 3B). In contrast, neuron specific enolase (NSE) was upregulated, indicative of a developing neuroendocrine phenotype. Neurite elongation was assessed by measuring neurite extensions between two adjacent cells at a number of time points during CSFBS or ENZ, up to 14 days with increased neurite length over time (Fig. 3C,D). We hypothesised that inhibition of Ca_V_1.3 with Nifedipine could affect neurite outgrowth; however, the drug had no effect when tested on cells treated with CSFBS (Fig. 3E) after 10 days and after 14 days where culture medium with/without the drug was replenished every 3 days. A live-cell labelling protocol was employed to determine whether Ca_V_1.3 was localised on the plasma membrane, in addition to the cytoplasm (Fig. 2C). This strategy was adopted to avoid using fixatives and permeabilization agents that would normally enable intracellular antibody binding. In both untreated and ENZ-treated LNCaP (7 days), there was clear Ca_V_1.3 surface expression (Fig. 3F).

Live-cell Ca^2+^-fluorescence imaging was then carried out on LNCaP and VCaP. In the absence of drugs/agonists, LNCaP were quiescent with no changes in intracellular [Ca^2+^] during the 400 s recordings when perfused with normal PSS. Stimulation with 60 mM K^+^ PSS containing the Ca_V_1.3. activator, Bay K 8644 (10 µM) (high K^+^/Bay K) to cause depolarization and activate Ca_V_1.3 channels, rarely evoked Ca^2+^-transients in LNCaP that exceeded the 0.2ΔF/F_0_ analysis threshold (Fig. 4A). In contrast, a proportion of cells that were cultured in CSFBS for 10 days exhibited Ca^2+^-transients when challenged with high K^+^/Bay K (Fig. 4B). The percentage of responding cells increased with CSFBS duration (up to 14 days, Fig. 4C). To determine whether the Ca^2+^-transients were due to Ca^2+^-influx via Ca_V_1.3 ion channels, cells were pre-treated with Nifedipine (1 μM) for 5 min prior to recordings—this significantly reduced the percentage of responding cells (Fig. 4D, 19.1 ± 2.1% in 10 day CSFBS to 1.8 ± 0.8% by Nifedipine, p < 0.001, N = 5 cultures). To further establish involvement of Ca_V_1.3 channels, siRNA knockdown was performed on LNCaP that were cultured in CSFBS (14 days). Successful *CACNA1D* knockdown to < 50% expression was obtained and confirmed with qPCR (Fig. 4E). It was not possible to also perform Western blots as the quantity of cells after CSFBS treatment is diminished; in knockdown experiments, cells were used in live-cell imaging and the remainder were used for qPCR validation. Compared with scrambled controls where Ca^2+^-transients could be evoked in 18.9 ± 6.8% of LNCaP, only 1.0 ± 0.5% of siRNA-transfected cells exhibited responses (p = 0.058, N = 3, Fig. 4F) supporting the hypothesis that after CSFBS culturing, Ca_V_1.3 channels were functionally expressed and mediated depolarization-evoked Ca^2+^-transients.

Cells were also studied with whole-cell patch-clamp electrophysiology to determine whether candidate voltage-gated Ca^2+^ channels e.g. Ca_V_1.3 were present. Cells were patched with Cs^+^-filled pipettes to block outward K^+^ currents which are dominant in this cell line^39^. A typical current–voltage protocol was used, where cells were held at − 60 mV, stepped to − 100 mV for 2 s to remove inactivation and then stepped to increasingly positive potentials up to + 50 mV to evoke currents. LNCaP did not exhibit detectable inward currents when patched in PSS or in modified Ba^2+^/TEA PSS, a solution containing TEA to block any residual K^+^-currents and using Ba^2+^ as the charge carrier instead of Ca^2+^. The latter approach is often used in electrophysiology as Ca_V_1.3 channels are more permeable to Ba^2+^ and therefore detectable, typically larger, non-inactivating currents can be obtained^40,41^. (Fig. 5A,B). A further strategy to enhance current detection was perfusion with divalent-free PSS, used to remove inactivation of Ca^2+^-channels^42^ that is caused by divalent ions in PSS e.g. Mg^2+^. Under this condition, small currents were detected (Fig. 5A,B).

Given the emergence of Ca_V_1.3-mediated Ca^2+^-transients in LNCaP that were cultured in CSFBS, these cells were also patch-clamped. Inward currents could be recorded from LNCaP in CSFBS cultured up to 25 days, (n = 11, N = 4 cultures) and were significantly larger than currents from untreated cells (Fig. 5C − 50 mV p < 0.01, − 40 mV p < 0.001, − 30 mV p < 0.01 and − 20 mV p < 0.05). These were often reduced by the Ca_V_3.2 blocker, mibefradil (0.3 μM, n = 6 cells, N = 4 cultures, Fig. 5D,E) and were Nifedipine-insensitive (1 μM). Occasionally, Nifedipine-sensitive currents were encountered in treated cells. Recordings from 3 cells from one culture, CSFBS for 14 days, showed inward currents that were reduced by nifedipine (1 μM) and largely recovered on washout (Fig. 5F,G). This finding is consistent with the higher-throughput Ca^2+^-imaging data where a subpopulation of cells fired Ca^2+^-transients during high K^+^/Bay K stimulation. In patch-clamp, the likelihood of successfully recording from a cell in this small subpopulation will be low; nevertheless, the data indicates that functional Ca_V_1.3 channels may be present in some LNCaP after CSFBS.

ENZ-treated LNCaP exhibited Ca^2+^-transients to high K^+^/Bay K 8644; after 4 days treatment (11.2 ± 3.1% cells responded, N = 3 cultures) and after 10 days (26.9 ± 5.0% cells responded, N = 3 cultures, Fig. 6A). Nifedipine significantly reduced the % of responding LNCaP (3.9 ± 1.2%, 10 days ENZ, p < 0.01, N = 3 cultures, Fig. 6B). In patch-clamp experiments, large, inward currents (divalent-free solution) were present in ENZ-treated (Fig. 6C) LNCaP that were Nifedipine-insensitive (4 days ENZ, n = 9 cells, N = 4 cultures, 1 μM, Fig. 6D and 14 days ENZ, n = 5 cells, N = 2 cultures, Fig. 6E,F). These had similar properties to voltage-dependent inward currents detected in CSFBS treated cells (Fig. 5).

VCaP (T2E^+^, overexpressing *CACNA1D*/Ca_V_1.3), were distinctively different from LNCaP with most cells tested exhibiting small inward currents in Ba^2+^/TEA PSS (Fig. 7A). These currents activated above − 50 mV and peaked around 0 mV, consistent with the properties of L-type currents (n = 7 cells, N = 5 cultures) Fig. 7B). Unexpectedly, the currents showed little sensitivity to Nifedipine but could be reduced by Ni^2+^ (100 μM) which typically blocks Ca_V_3.2 currents. The current–voltage plot of the residual current in the presence of Ni^2+^ was shifted to the right by 20 mV (n = 7, N = 5 cultures) and may represent a Ca_V_1.3 current; however, this was not further elucidated. Currents in divalent-free solution were markedly larger than in Ba^2+^/TEA PSS and were similar to currents recorded from ENZ or CSFBS treated LNCaP (n = 9, N = 7 cultures) (Fig. 7C).

In contrast to LNCaP, some VCaP exhibited spontaneous Ca^2+^ transients or oscillations (Fig. 7D), this was calculated to be a property of 2.9 ± 1.2% of untreated VCaP (N = 5 cultures). High K^+^/Bay K elicited Ca^2+^-transients in 16.8 ± 2.6% of cells (N = 4 cultures, Fig. 7E). ENZ enhanced the percentage of responding cells to 41.4 ± 7.6% at 14 days (p < 0.01, N = 4 cultures), moreover, these were nifedipine-sensitive (Fig. 7F). The amplitudes of Ca^2+^-transients were unaffected by ENZ (Fig. 7G).

## Discussion

This study found high Ca_V_1.3 expression in PCa public datasets, a human PCa TMA and across a cell panel compared with normal tissue/cells. Ca_V_1.3 localisation at the plasma membrane (in addition to the cytoplasm) in untreated or hormone-therapy mimicking conditions suggested that canonical Ca^2+^-influx function could be operational. CSFBS/ENZ enhanced *CACNA1D* expression whereas total protein expression and plasma membrane localisation were unchanged. Physiological evidence for Ca_V_1.3 functioning as ion channels after CSFBS/ENZ in T2E^– ^LNCaP and in untreated or treated T2E^+^-VCaP from live-cell Ca^2+^-imaging was supported by detection of voltage-gated Ca^2+^-currents in electrophysiology.

*CACNA1D* overexpression in PCa has been reported previously^17,18,22,30^ and our analysis of large TCGA datasets demonstrated high *CACNA1D* expression across a range of cancers. Of the 17 cancers in the dataset, *CACNA1D* expression was greatest in PCa, suggesting contribution to the development, progression, or treatment response of PCa. Overexpression of *CACNA1D* was detected in primary and metastatic PCa, across all Gleason grades. Immunohistochemistry of our TMA^32^ revealed Ca_V_1.3 protein overexpression in tumour vs non-tumour tissue, consistent with another report^14^.

*CACNA1D* expression was highest in androgen-sensitive cells, particularly VCaP which harbours an AR-amplification, also shown in our Western blot data^43^ and is T2E^+^^44^. Interestingly, while Western blots showed similar Ca_V_1.3 expression across most of the cell panel, it was markedly higher in VCaP. This is consistent with the T2E fusion gene causing overexpression of ERG and the ERG-related *CACNA1D*, an observation that has also been reported in large patient datasets^17^. To date, evidence for Ca_V_1.3 function in VCaP has not been shown using electrophysiological or live-cell Ca^2+^-imaging techniques.

We hypothesised that Ca_V_1.3 would be localised on the plasma membrane, on the basis that Ca_V_1.3 is a voltage-dependent ion channel, normally enabling Ca^2+^-influx^12^. This was supported in live-labelling of non-permeabilised LNCaP which unmasked plasma membrane localisation, suggesting that some Ca_V_1.3-fluorescence could represent membrane ion channels. Immunofluorescence of fixed, permeabilized cells also showed extensive cytoplasmic Ca_V_1.3. VCaP exhibited nuclear Ca_V_1.3-fluorescence, unexpectedly as a large protein (220–250 kDa) is unlikely to be able to enter via nuclear pores. One interpretation is that Ca_V_1.3 has a non-canonical function in VCaP, acting perhaps as a transcription factor, as has been reported for other ion channels in cancer cells including the KCNQ/Kv7.1 channel^45^. As our antibody targets an extracellular region (ACC-311, Manufacturer states amino acid residues 215–227, second extracellular loop, repeat I), the explanation seems unlikely to involve a c-terminus cleavage product, typical of Ca_V_1.3, that translocates to the nucleus^46^. Interestingly, nuclear Ca_V_1.3 expression was detected in HCT116 colon cancer cells using the same antibody^16^ as in the present study. Other studies support ion channel expression on the nuclear membrane including K_V_1.1, K_V_1.2, K_V_1.3 and K_V_2.2^47^ that may regulate nuclear membrane potential or modulate transcription factors; further work is required to understand nuclear expression of Ca_V_1.3 in VCaP.

The present study focused on androgen-sensitive cell models, and as these had highest *CACNA1D*/Ca_V_1.3 expression, we were particularly interested in Ca_V_1.3 expression and function during the initial response to CSFBS/ENZ. Ca_V_1.3 blockade by Nifedipine concentration-dependently inhibited proliferation of LNCaP (and C4-2 and C4-2B) suggesting that Ca_V_1.3 may contribute to proliferation of some androgen-sensitive PCa cells. In contrast, Nifedipine (1 μM) did not affect growth of neurite-like extensions of CSFBS-treated LNCaP, suggesting that this property may not require Ca_V_1.3-mediated Ca^2+^-influx. Others have shown that Ca_V_3.2 blockade reduces neurite growth although does not fully inhibit it^48–51^. We cautiously interpret the apparent lack of effect of Nifedipine in the neurite experiments over a timecourse of 14 days in which media and fresh nifedipine was replaced every 3 days. It is possible that the nifedipine effect diminished during incubation and Ca_V_1.3 channels regained activity, this could be investigated in the future using stable Ca_V_1.3 knockdown models.

While *CACNA1D* was enhanced in LNCaP after CSFBS/ENZ, this was not matched with increased Ca_V_1.3 expression. The impact of increased *CACNA1D* expression during PCa response to hormone-therapy is not understood and may contribute to transcriptional regulation of genes governing cell survival mechanisms during the androgen-sensitive phase of treatment. This was not explored further in the present study. The finding that under these conditions, functional Ca_V_1.3 channels could be detected (see below) might be explained by effects on auxiliary α2δ or β subunits whose co-expression with the pore-forming α1 subunit increases channel expression and voltage-dependence of both activation and inactivation^12^. Androgen deprivation might also promote enhanced trafficking to the plasma membrane; however, contributory mechanisms are currently unknown. Increased Ca^2+^-influx via voltage-gated channels in PCa might govern gene expression, enzymatic activity, secretion, protein phosphorylation/dephosphorylation or glucose metabolism^52^. Dysregulated Ca^2+^-signalling in cancer is associated with many of the cancer hallmarks; importance is given to understanding the kinetic properties of the Ca^2+^-signal and how this is decoded by the cell^53^.

Two methodologies were employed here to assess whether Ca_V_1.3 had ion channel function in androgen-sensitive PCa cells; live-cell Ca^2+^-imaging and patch-clamp electrophysiology. LNCaP (non-amplified AR, wild-type AR, T2E^−^) and VCaP (amplified AR, AR-V7 mutated, T2E^+^) were used, enabling broad comparison of molecular phenotypes. These techniques enable ion channel function to be directly evaluated in real-time. In Ca^2+^-recordings, LNCaP were quiescent in the absence of stimulation, displaying little baseline activity, similar to another report^54^, in comparison to VCaP in which a small population (around 3%) exhibited baseline Ca^2+^-oscillations. LNCaP rarely responded to Ca_V_1.3 activation via depolarization/Ca_V_1.3 activator (high K^+^/Bay K); however, VCaP were more likely to fire Ca^2+^-transients when stimulated. This aligns with overexpression of *CACNA1D* and Ca_V_1.3 in T2E^+^-VCaP.

Depolarization rarely evoked detectable inward currents in LNCaP; in contrast, small inward currents could be reliably recorded from VCaP. These activated positive to − 50 mV, peaked at 0 mV, typical for Ca_V_1.3 channels and were partially sensitive to Ni^2+^, indicating a component of Ca_V_3.2 channels although L-type channels also have some sensitivity to Ni^2+^^55^. The concentration of Ni^2+^ used here, 100 μM, is less than the IC_50_ (324 μM) reported for L-type channels in urethral smooth muscle so here, its effect may represent Ca_V_3.2 inhibition. The Ni^2+^-resistant current–voltage plot was consistent with Ca_V_1.3, showing a 20 mV rightward shift in peak current voltage. To our knowledge, this is the first electrophysiological study of VCaP, or of T2E^+^ PCa cells. Divalent-free Hanks was then used to enhance current amplitude and enable further characterisation^42^. LNCaP currents remained small; however, VCaP exhibited large inward currents which activated from − 70 mV and peaked at − 50 mV. The observation that removal of divalent ions from the extracellular solution significantly enhanced current amplitude, indicated that divalent ions caused channel inactivation. These conditions were then used to study LNCaP currents in the remainder of the study.

Compelling evidence for Ca_V_1.3 functional expression in LNCaP and VCaP was obtained from live-cell Ca^2+^-imaging experiments. After CSFBS/ENZ, a subpopulation of LNCaP exhibited Ca^2+^-transients upon the Ca_V_1.3 activation stimulus. This subpopulation increased with increased duration of CSFBS/ENZ, up to 14 days, representing the initial response to hormone therapy prior to development of treatment resistance. The finding that nifedipine reduced the percentage of cells exhibiting Ca^2+^-transients after stimulation to around 1%, strongly suggests that the events were mediated by Ca_V_1.3 channels. Further evidence is seen in CSFBS-LNCaP transfected with siRNA to knockdown *CACNA1D* where the percentage of cells responding to the stimulation was reduced to 1%, corresponding with more than 50% gene reduction. Similar to another report, *CACNA1D* silencing in our hands was challenging^22^ and extensive optimisation was required to achieve *CACNA1D* knockdown. These important experiments provide novel data, independent of pharmacology, supporting enrichment of LNCaP expressing functional Ca_V_1.3 channels in CSFBS or ENZ. VCaP, which have baseline oscillations, Ca_V_ currents and exhibit Ca^2+^-transients following Ca_V_1.3 stimulation, were also more likely to be responsive following ENZ, with more than double the percentage of responsive cells. Ca^2+^-transients in both untreated and ENZ-VCaP were nifedipine-sensitive, strongly supporting Ca_V_1.3 function, consistent with the Ni^2+^-resistant inward currents detected in untreated VCaP.

Another key finding was the increased likelihood of recording measurable voltage-dependent inward currents after CSFBS/ENZ. These currents were frequently sensitive to the Ca_V_3.2 blocker mibefradil and less frequently, currents were blocked by 1 μM Nifedipine. We propose that automated patch-clamp recordings of markedly greater number of cells per culture is needed to further investigate this; particularly as Ca^2+^-imaging showed around 20–30% of cells with Ca_V_1.3 function. Our findings are consistent with Mariot et al. who detected a threefold increase in the percentage of LNCaP with Ca_V_3.2 currents after culture in cAMP analogues or CSFBS to induce neuroendocrine differentiation^51^.

The apparent Nifedipine-insensitivity of the majority of inward currents recorded from LNCaP warrants further consideration, particularly as the recording conditions are different from the normal Hanks’ (PSS) solution used in Ca^2+^-imaging protocols. Recordings using 10 mM Ba^2+^-Hanks' may have reduced sensitivity to Nifedipine as is reported for vascular myocytes where Ca_V_1.2 currents were abolished by Nifedipine when 2 mM Ba^2+^-solution was used, but in 10 mM Ba^2+^ a Nifedipine-insensitive component was recorded^56^. Furthermore, the absence of Ca^2+^ in our divalent-free solution may have impeded the action of Nifedipine as it is reported that dihydropyridines (such as nifedipine) require the presence of Ca^2+^ ions which act as allosteric modulators^56–58^. Finally, Ca_V_1.3 is less sensitive to Nifedipine than Ca_V_1.2 and the concentration used here (1 μM) may have been insufficient to achieve inhibition of the currents ^59^. Taken together, this may suggest that our recording conditions, chosen to enhance Ca_V_ current amplitude, impeded the ability of Nifedipine to bind to Ca_V_1.3 and bring about full channel blockade.

Our findings reveal distinctive Ca^2+^-signalling properties between LNCaP and VCaP with the latter having baseline oscillations and inward currents prior to treatment conditions. After CSFBS or ENZ, while a higher proportion of LNCaP expressed functional Ca_V_1.3 activity, there was little evidence of oscillations. The relevance of these different Ca^2+^-signalling profiles is not yet known but it has been hypothesised that sustained Ca^2+^-increase via influx may drive proliferation whereas increased oscillation frequency may alter activation of Ca^2+^-dependent transcription factors^53^. Ca_V_1.3 activity cannot be considered in isolation but as one component of myriad Ca^2+^-channels, transporters and pumps that collectively regulate the spatial–temporal nature of Ca^2+^-signalling.

## Conclusions

This study presents novel data on emerging functional Ca_V_1.3 channels in T2E-, androgen-sensitive LNCaP under hormone-therapy mimicking conditions. Involvement of other mechanisms, including Ca_V_3.2 is also supported. The T2E^+^ VCaP model was found to have higher baseline Ca^2+^-activity demonstrated through inward Ca_V_3.2 currents, Ca^2+^-oscillations and depolarization-evoked-Ca^2+^-transients (Ca_V_1.3), the latter was enhanced in hormone-therapy conditions. The cellular decoding of these Ca^2+^-signals during the initial response to treatment whether contributing to cell death, survival or altered gene transcription remains to be elucidated.

## Supplementary Information

Supplementary Figures.

## Data Availability

Data is available from the corresponding author on reasonable request. Publicly available data from the Human Protein Atlas was used in Fig. 1A and can be found at www.proteinatlas.org/ENSG00000157388-CACNA1D/pathology (v21.1.proteinatlas.org). Publicly available data from the Memorial Sloan-Kettering Cancer Center (MSKCC) was used in Fig. 1B,C and can be found at www.ncbi.nlm.nih.gov/geo/query/acc.cgi?acc=GSE21032.
