# Supplementary material for: CACNA1D overexpression and voltage-gated calcium channels in prostate cancer during androgen deprivation
Source: Sci Rep. 2023 Mar 22;13:4683. doi: 10.1038/s41598-023-28693-y (PMC10033880; doi:10.1038/s41598-023-28693-y)
Supplement: Supplementary file 1 — Supplementary Figures. [file 41598_2023_28693_MOESM1_ESM.docx]

**
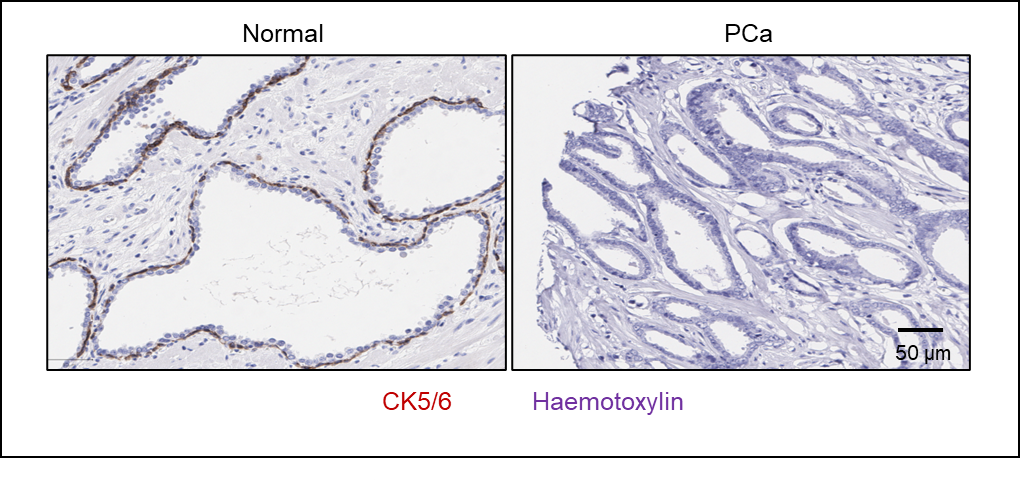
**

**Supplementary Figure S1: CK5/6 staining in normal and prostate cancer tissues​**

Immunohistochemistry was performed on an in-house TMA microarray containing PCa tumour biopsies and matched normal/benign tissues, obtained from the NI Biobank. Tissues were independently assessed by a pathologist where CK5 staining, and gland appearance criteria were applied to differentiate tumour tissue from normal/benign regions. Micrographs demonstrate low CK5/6 staining which was typically observed in tumour tissues compared to normal/benign tissue.


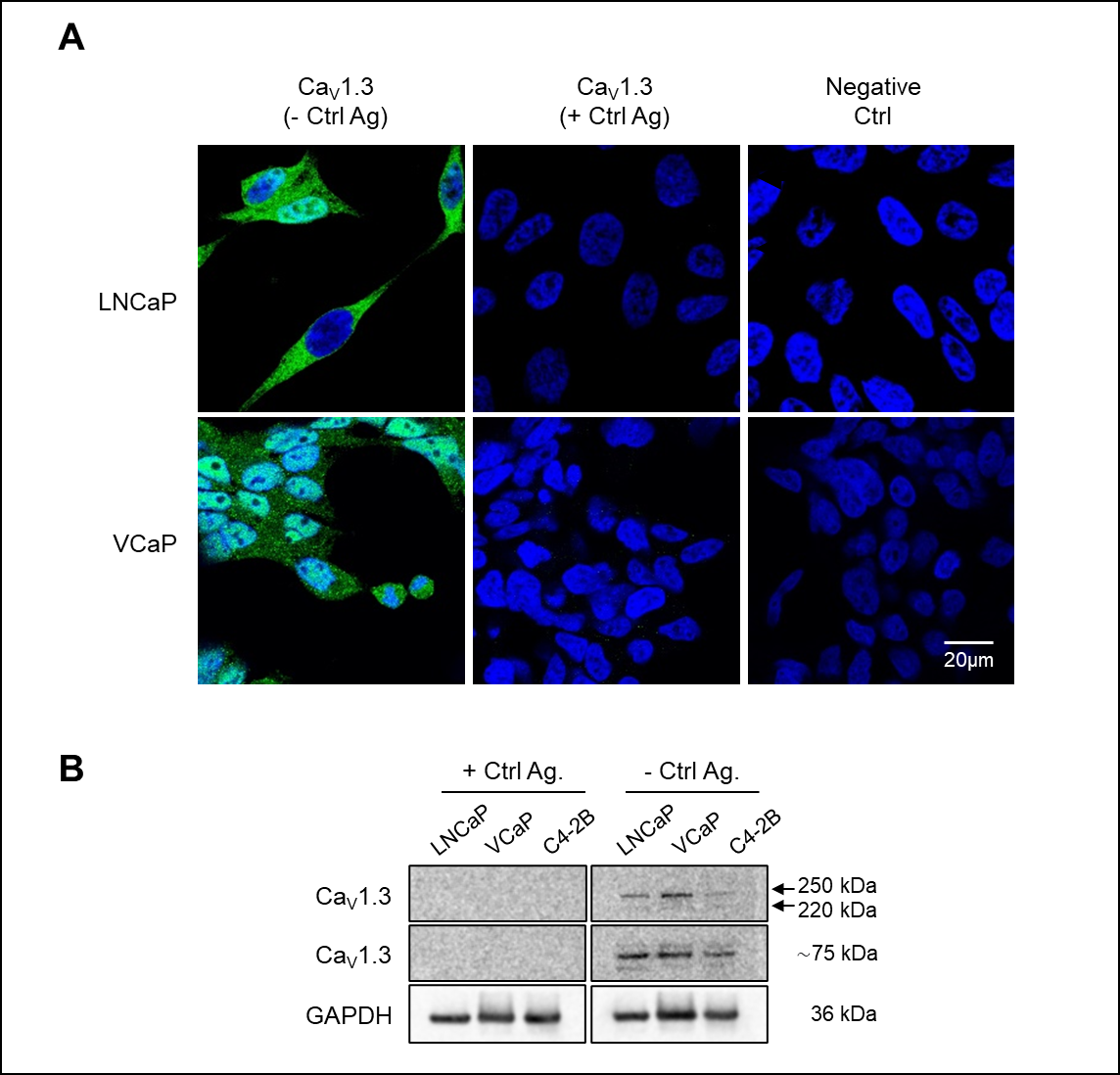


**Supplementary Figure S2: Validation of specificity and molecular weight using anti-Ca_V_1.3 control immunogen and secondary-only controls**

**A.** Antibody validation of Ca_V_1.3 (ACC-311, Alomone) was performed using a peptide control antigen (BLP-CC311). Immunofluorescence micrographs demonstrated Ca_V_1.3-positive labelling under normal conditions (left panel) and the absence of fluorescence when the Ca_V_1.3 antibody had been pre-incubated with peptide control antigen (middle panel) in LNCaP and VCaP (N=3). A representative micrograph of negative (secondary-only) control is shown in the right panel. **B.** Western blots demonstrating chemiluminescence resolution of Ca_V_1.3 bands in LNCaP, VCaP and C4-2B protein lysates, in the absence of peptide control antigen (Ag) or with the primary antibody pre-incubated with the peptide control antigen (N=3).​​ Uncropped blots are shown in Supplementary Figure S3.


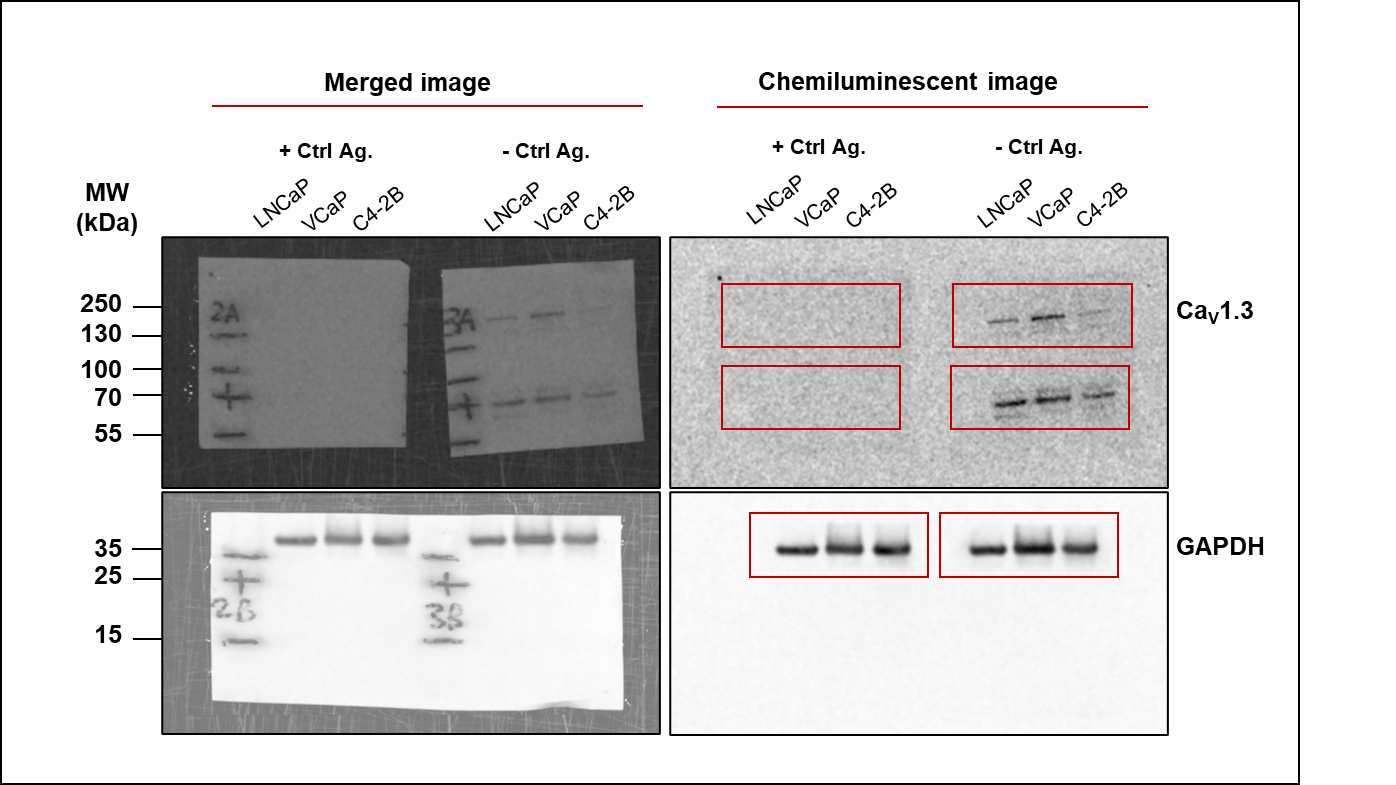


**Supplementary Figure S3: Full-length original blots relating to Supplementary Figure S2B**

Full length membrane is shown from original images of aligned cut blots. Blots were cut prior to hybridisation with antibodies. Protein(s) of interest are labelled to the right of each image and relevant bands are indicated with red boxes. The molecular weight (MW, units: kDa) is indicated on the left of blot image in conformity with protein ladder markers. Merged images are shown to the left of chemiluminescent western blot images to visualise protein ladder and membrane boundaries.


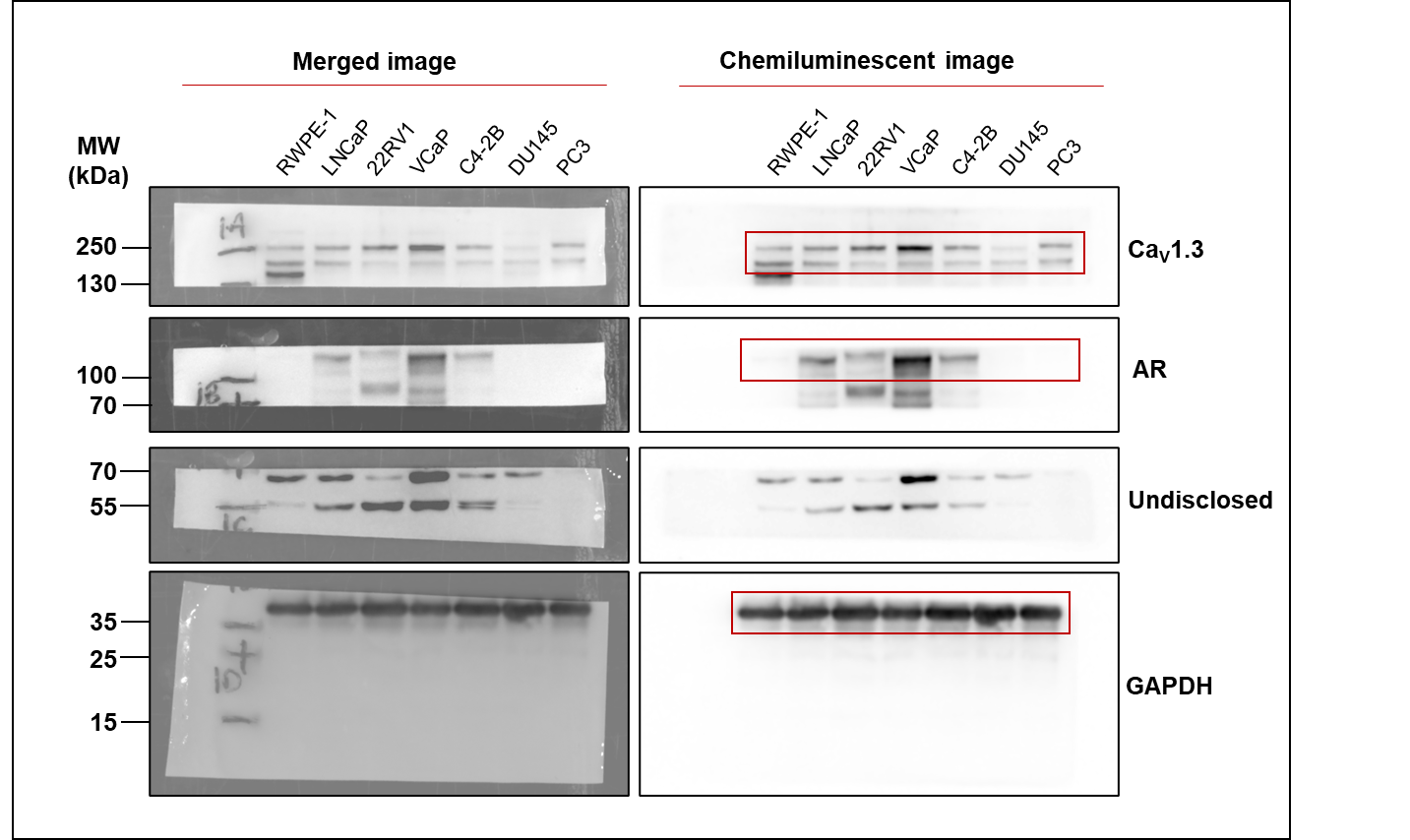


**Supplementary Figure S4: Full-length original blots relating to Figure 2B Prostate Cell lines**

Original images of cut blots were aligned to show the full length membrane. Blots were cut prior to hybridisation with antibodies. The protein band(s) of interest are recorded to the right of each blot image. The molecular weight (MW, units: kDa) representing each marker of the protein ladder are shown on the left of images. The relevant protein bands are indicated with red boxes on the chemiluminescent images of membrane. Protein bands irrelevant to manuscript are labelled as “Undisclosed”. Merged images (M) are shown to demonstrate the protein ladder marker and membrane boundary.


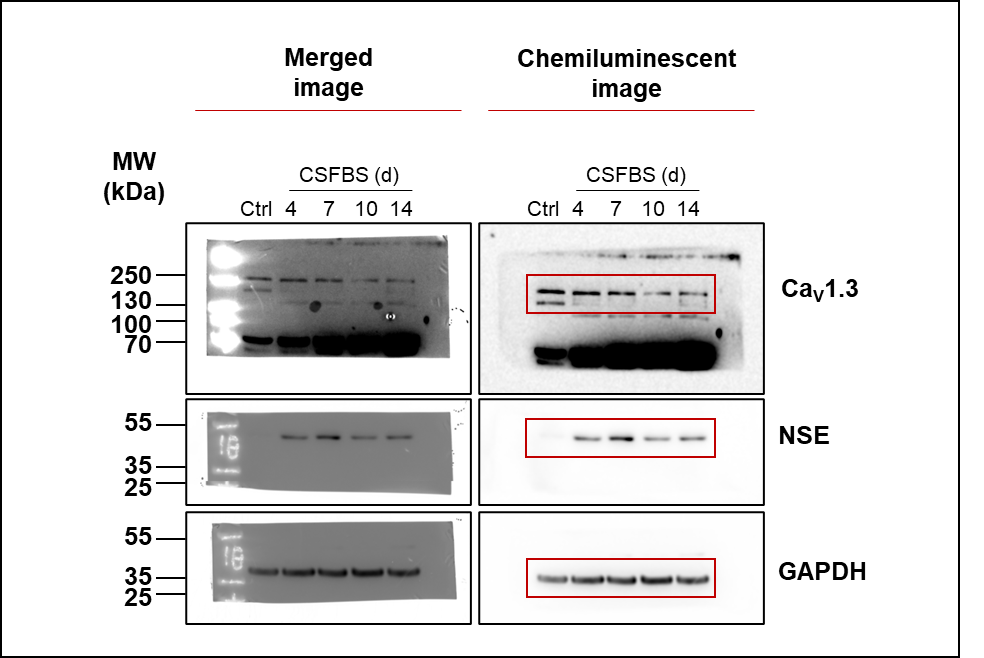


**Supplementary Figure S5: Full-length original blots relating to Figure 3B (CSFBS, left panel)**

Representation of full-length membrane is shown from original images of aligned cut blots. Five lanes were used in SDS-PAGE and transferred to nitrocellulose membrane. Blots were cut prior to antibody hybridisation. Protein(s) of interest are indicated to the right of blot images where relevant bands are highlighted with red boxes. The molecular weight (MW, units: kDa) of protein ladder markers are shown on the left of images. Blots were stripped and re-incubated to target alternative proteins (i.e. NSE membrane was stripped and re-incubated with anti-GAPDH). Merged images are shown on the left of chemiluminescent images, for visualisation of the protein ladder and membrane boundaries.

**
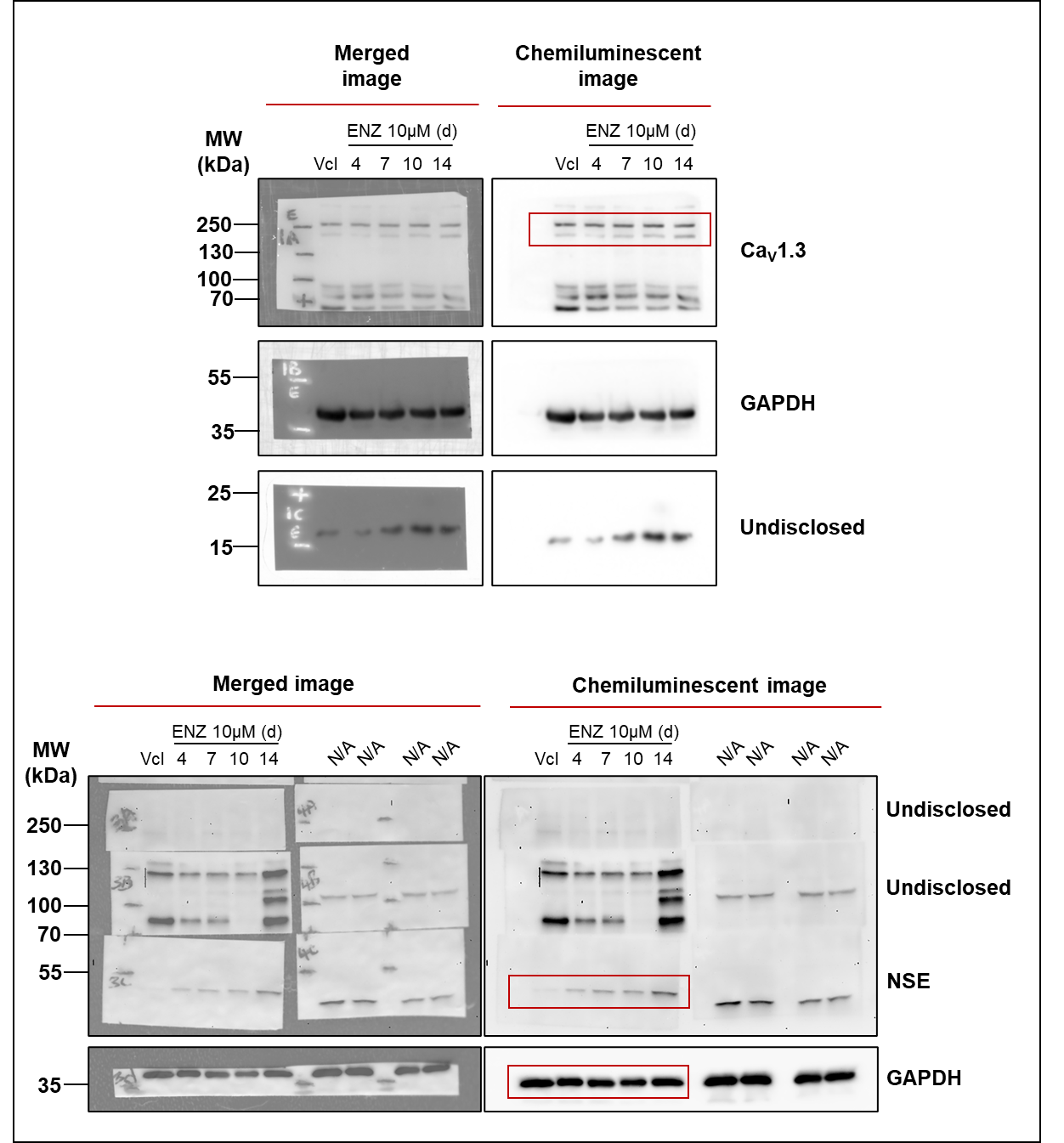
**

**Supplementary Figure S6: Full-length original blots relating to Figure 3B (ENZ, right panel)**

Representation of full-length membranes from original cut blots. Blots were cut prior to antibody hybridisation. The protein(s) of interest are outlined to the right of each membrane image with protein ladder molecular weights (MW, units: kDa) shown on the left. The protein bands(s) of interest are indicated with red boxes. In upper blot, five lanes were used in SDS-PAGE before membrane transfer and shows Ca_V_1.3 and related GAPDH expression during ENZ treatment of LNCaP cells. Lower blot represents NSE and GAPDH protein expression during ENZ treatment of LNCaP cells. Unrelated lysate lanes are denoted as not applicable (N/A) above membrane images. Protein bands irrelevant to manuscript are labelled as "Undisclosed”. Merged images (M) are used for visualisation of the protein ladder and membrane boundaries.
